# Identification of Salivary Metabolic Signatures Associated with Primary Sjögren’s Disease

**DOI:** 10.3390/molecules28155891

**Published:** 2023-08-05

**Authors:** Addy Alt-Holland, Xuejian Huang, Tatiana Mendez, Mabi L. Singh, Athena S. Papas, Joseph Cimmino, Tiffany Bairos, Elizabeth Tzavaras, Elizabeth Foley, Sarah E. Pagni, James D. Baleja

**Affiliations:** 1 Department of Endodontics, Tufts University School of Dental Medicine, One Kneeland Street, Boston, MA 02111, USA; 2 Tufts University School of Dental Medicine, One Kneeland Street, Boston, MA 02111, USA; 3 Program in Pharmacology and Drug Development, Tufts University Graduate School of Biomedical Sciences, 136 Harrison Avenue, Boston, MA 02111, USA; 4 Department of Diagnostics Sciences, Division of Oral Medicine, Tufts University School of Dental Medicine, One Kneeland Street, Boston, MA 02111, USA; 5 Department of Public Health and Community Service, Division of Biostatistics and Experimental Design, Tufts University School of Dental Medicine, One Kneeland Street, Boston, MA 02111, USA; 6 Department of Developmental, Molecular and Chemical Biology, Tufts University School of Medicine, 136 Harrison Avenue, Boston, MA 02111, USA; 7 Department of Medical Education, Tufts University Graduate School of Biomedical Sciences, 136 Harrison Avenue, Boston, MA 02111, USA

**Keywords:** Sjögren’s disease, saliva, biomarkers, metabolomics, nuclear magnetic resonance spectroscopy

## Abstract

Sjögren’s disease (SjD) is the second most prevalent autoimmune disorder that involves chronic inflammation of exocrine glands. Correct diagnosis of primary SjD (pSjD) can span over many years since disease symptoms manifest only in advanced stages of salivary and lachrymal glandular destruction, and consensus diagnostic methods have critical sensitivity and selectivity limitations. Using nuclear magnetic resonance (NMR) spectroscopy, we determined the composition of metabolites in unstimulated saliva samples from 30 pSjD subjects and 30 participants who do not have Sjögren’s disease (non-Sjögren’s control group, NS-C). Thirty-four metabolites were quantified in each sample, and analysis was conducted on both non-normalized (concentration) and normalized metabolomics data from all study participants (ages 23–78) and on an age-restricted subset of the data (ages 30–70) while applying false discovery rate correction in determining data significance. The normalized data of saliva samples from all study participants, and of the age-restricted subset, indicated significant increases in the levels of glucose, glycerol, taurine, and lactate, as well as significant decreases in the levels of 5-aminopentanoate, acetate, butyrate and propionate, in subjects with pSjD compared to subjects in the NS-C group. Additionally, a significant increase in choline was found only in the age-restricted subset, and a significant decrease in fucose was found only in the whole study population in normalized data of saliva samples from the pSjD group compared to the NS-C group. Metabolite concentration data of saliva samples from all study participants, but not from the age-restricted subset, indicated significant increases in the levels of glucose, glycerol, taurine, and lactate in subjects with pSjD compared to controls. The study showed that NMR metabolomics can be implemented in defining salivary metabolic signatures that are associated with disease status, and can contribute to differential analysis between subjects with pSjD and those who are not affected with this disease, in the clinic.

## 1. Introduction

Sjögren’s disease is a progressive autoimmune disease that affects 0.1–0.8% of the population in the US, with similar prevalence worldwide [1,2,3,4]. This a poorly defined range that reflects the difficulty in diagnosis due to a lack of uniform diagnostic criteria, and an overlap of primary Sjögren’s disease (pSjD) with other autoimmune rheumatic disorders can also occur [1,4]. Since Sjögren’s is a well-characterized, systemic disease, the term Sjögren’s syndrome has been replaced with the term Sjögren’s disease in recent years. This change reinforces the serious, systemic nature of this disease for both healthcare providers and patients. The disease affects mainly women (9:1 female/male ratio) [2,3], with an onset typically between 40–60 years [4,5,6]. SjD is characterized by chronic inflammation of exocrine glands that leads to progressive destruction of salivary and lachrymal glands. The clinical symptoms of SjD include dry mouth and dry eyes, pain, and disabling fatigue that affect nearly all patients and can result in anxiety and depression. In a subset of patients, hyperactivity of CD4 T cells, memory cells, B cells, and plasma cells will severely impair multiple organ systems, including muscles, joints, nerves, and kidney. This can result in functional disability and reduced quality of life [7]. Increased B-cell activity in pSjD is linked to an increased risk of malignant transformation, such as the development of lymphoma [8,9]. Extraglandular manifestations are included in the ESSDAI, the EULAR Sjögren’s Syndrome Disease Activity Index, which comprises 12 domains: constitutional, lymphatic, glandular, vascular and dermal, musculoskeletal, pulmonary, renal, central and peripheral nervous system, hematologic, and immunologic systems. This instrument was developed by the consensus of worldwide experts from European and North American countries and is used as an outcome criterion to evaluate systemic disease activity in pSjD clinical studies and daily practice.

Early diagnosis of pSjD, and in turn, early treatment for mucosal signs and dryness symptoms, is key in inhibiting complications from the progression of the disease. The challenge of correct diagnosis of pSjD stems from the long duration between disease initiation to its diagnosis, and the sensitivity and specificity of conventional, consensus diagnostic methods [10]. The time from the onset of symptoms to a diagnosis is, on average, over 5 years as symptoms manifest only in advanced stages of glandular destruction. Due to delayed diagnosis, complications from disease progression may irreversibly damage the soft and hard tissue of the mouth and exocrine glands in the esophagus, stomach, bowel, bladder, and pancreas [7]. As per the criteria for SjD by the American-European Consensus Group [11,12], in addition to the objective measures of reduced saliva and tears secretion, diagnosis criteria for SjD requires the presence of serologic SS-A auto-antibodies and positive pathological findings from labial minor salivary gland biopsy (MSGB). Both methods comprise significant drawbacks. Although anti-SSA positive serology is considered a hallmark of the disease, only 70% of SjD patients are positive for the SS-A blood marker [13]. MSGB, commonly referred to as the “lip biopsy”, is considered as the “gold standard” of pSS diagnosis [14], and has an estimated sensitivity and specificity between 78% to 86%, respectively, depending on the assessment method [15,16]. This surgical procedure is uncomfortable for the patient and is associated with complications that range from temporary numbness, pain, bleeding, bruising and wound infection to a long-term complication of paresthesia of the lower lip [14,15,16].

Multiple studies have emphasized the necessity of alternative biomarkers to pSjD, including salivary gland ultrasonography [17,18], serologic antigens and autoantibodies [19,20,21], and proteomics [22,23,24,25]. Saliva is a rich and accessible oral fluid that contains real-time information about the physiological condition of the body. Salivary biomarkers reflect both local, oral disorders as well as distant pathological changes in distal tissues and organs [26,27]. Whereas studies have focused on saliva proteomic, transcriptomic, and genomic biomarkers for pSjD [28,29], sensitive and specific salivary biomarkers for this disease have not yet been available. Metabolic profiling (metabolomics) of biofluids and tissues is a powerful diagnostic tool for gaining insights into the biology, toxicology and development of multiple pathologies [30]. It is appreciated as the science closest to early diagnosis of disease, even before the appearance of disease symptoms [31], and is applicable for a wide variety of biological samples including blood, urine, saliva, cells, and extracts from tissue and organ biopsies. Metabolomics applications are evident in diagnostics of cancer, metabolic and neurodegenerative disorders, and rheumatic and pulmonary diseases [30,32,33,34]. Salivary metabolomics is pertinent for diagnosis of oral cancers, periodontal, autoimmune, and metabolic diseases [35,36,37,38,39,40,41], and has revealed potential new biomarkers for SjD [42,43,44,45,46,47,48]. Changes in the expression of salivary metabolites may reflect injury of salivary glands due to chronic sialadenitis and increased oxidative activity that can be seen in autoimmune diseases, such as systemic lupus erythematosus [42,43]. The main analytical techniques employed in metabolomics are nuclear magnetic resonance (NMR) spectroscopy and mass spectrometry (MS). Both methods provide information on the relative and absolute concentrations of different classes of metabolites in a single measurement [49,50].

The aim of the study was to determine the composition and quantity of metabolites in unstimulated saliva samples from 30 pSjD subjects and 30 non-Sjögren’s control participants using ^1^H-NMR spectroscopy in order to identify differentially expressed metabolites that are associated with pSjD. Implementing false discovery rate correction, an analysis was conducted on both non-normalized (metabolite concentrations) and normalized salivary metabolomics data from all study participants, and, since pSjD is mostly age dependent, an age-restricted subset of data of samples from 30–70-year-old subjects in these two study groups. Compared to the controls, the saliva samples of pSjD subjects showed a significant increase in glucose, glycerol, taurine, and lactate as well as a significant decrease in 5-aminopentanoate, acetate, butyrate, and propionate in the normalized data from all study participants and in the age-restricted subset. Normalized data of saliva samples from the pSjD group compared to the NS-C group also revealed a significant increase in choline in the age-restricted subset of data, and a significant decrease in fucose in the whole study population. Metabolite concentration data of saliva samples from all study participants, but not the age-restricted subset, also indicated the significant increase in the levels of glucose, glycerol, taurine, and lactate in subjects with pSjD compared to controls. Thus, salivary metabolic profiles generated by NMR can potentially serve as a non-invasive approach for differential analysis of pSjD patients.

## 2. Results

### 2.1. Study Population Characteristics

**Figure 1:**
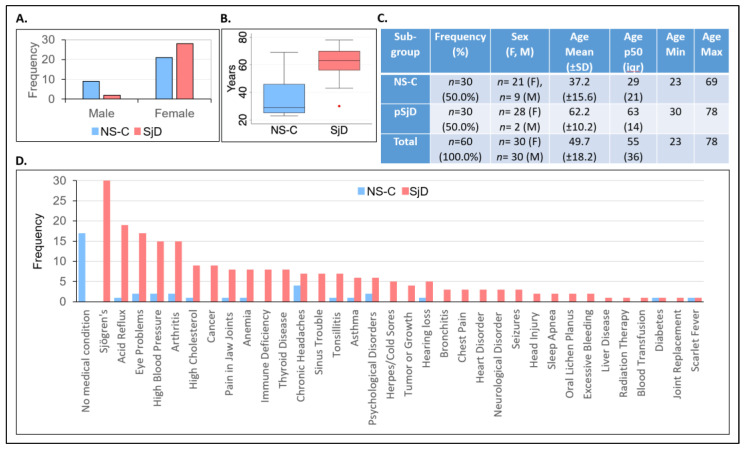
Distribution of sex, age, and medical conditions in the study population. The pSjD group (*n* = 30) was composed of 28 females and 2 males, while the NS-C group (*n* = 30) was composed of 21 females and 9 males (**A**). The median (p50) ages of the pSjD group and the NS-C group were 63 (iqr = 14), and 29 (iqr = 21), respectively (**B**). The red dot below the box plot represents an outlier data point outside of the iqr. Descriptive statistics of number, sex and age of subjects in the pSjD and NS-C groups (**C**). Participants reported their medical conditions using a standardized Medical History form (**D**). NS-C, non-Sjögren’s control group (blue bars); SjD, Sjögren’s disease group (red bars); *n*, sample size; F, female; M, male; SD, standard deviation; p50, median; iqr, interquartile range, min, minimum; max, maximum.

The study was open to both females and males, ages 18–80 years old, from all races and ethnicities, to allow for a wide range and diverse snapshot of subjects with primary Sjögren’s disease (pSjD) and non-Sjögren’s controls (NS-C) who visit our clinics. A total of 49 females and 11 males were enrolled in this single-center, cross-sectional pilot research study, of whom 43 (71.7%) were White, eight (13.3%) were Asian, three (5.0%) were African American, two (3.3%) were American Indian/Alaska Native, one (1.7%) was more than one race, and three (5%) did not report their race. All study participants strictly met the inclusion and exclusion criteria of the study. Individuals with pSjD had a controlled condition without any features contraindicative to participation. Non-Sjögren’s control (NS-C) participants did not have a history of any type of autoimmune disease (e.g., Sjögren’s, rheumatoid arthritis, and systemic lupus erythematosus). Medical conditions such as narcolepsy, Alzheimer’s disease or other diagnosed dementia, a psychotic disorder, and undergoing chemotherapy or use of any investigational drugs or participation in a clinical trial within 3 months of the study visit, were part of the exclusion criteria for both study groups. Within the primary Sjögren’s disease (pSjD) group, the disease was confirmed based on sero-positivity for anti-SSA antibodies by medical notes (*n* = 15, 50%), oral pathology report confirming lip biopsy (*n* = 10, 33.3%), both antibodies and lip biopsy (n = 3, 10%), or a physician letter (*n* = 2, 6%). The distribution of sex and median ages of subjects in the pSjD group (*n* = 30) and the non-Sjögren’s control group (NS-C, *n* = 30) are illustrated in Figure 1.

The pSjD group included two males (6.7%) and 28 females (93.3%), whereas the NS-C group included nine males (30.0%) and 21 females (70.0%) (Figure 1A). The sex of the subjects was significantly associated between groups (*p* < 0.02). The difference in the median age between the two groups was significant (*p* < 0.001), with a median age of 63 (interquartile range = 14) in the pSjD group, and a median age of 29 years (interquartile range = 21) in the NS-C group (Figure 1B,C). Demographics and baseline health characteristics of study participants are summarized in Supplementary Table S1.

Based on defined exclusion and inclusion criteria, subject screening, and report of medical history conditions, no subject in the NS-C group was diagnosed with Sjögren’s as opposed to, by design, all subjects reported Sjögren’s diagnosis in the pSjD group (*p* < 0.001). Sjögren’s disease is associated with multiple comorbidities that are included in the EULAR Sjögren’s Syndrome Disease Activity Index (ESSDAI) instrument. In the pSjD study group, Sjögren’s was significantly associated with acid reflux (*p* < 0.001), eye problems (*p* < 0.001), high blood pressure (*p* < 0.001), arthritis (*p* < 0.001), high cholesterol (*p* = 0.006), cancer (*p* = 0.002), pain in jaw joints (*p* = 0.03), anemia (*p* = 0.03), immune deficiency (*p* = 0.005), thyroid disease (*p* = 0.005), sinus trouble (infection, inflammation or other issues) (*p* = 0.01), tonsillitis (*p* = 0.05), and herpes/cold sores (*p* = 0.05) conditions in comparison to the NS-C group (Figure 1D, and Supplementary Table S1). As expected from the general population that visit our dental clinics, while some study participants in the NS-C group reported different medical conditions, such as chronic headaches, seventeen subjects (56.7%) in that group reported no medical conditions (*p* < 0.001). Treemap charts of the distribution of medical conditions reported in the pSjD and NS-C groups of the study population are provided in Supplementary Figure S1.

### 2.2. Metabolomics and Statistical Analysis of All Saliva Samples

**Figure 2:**
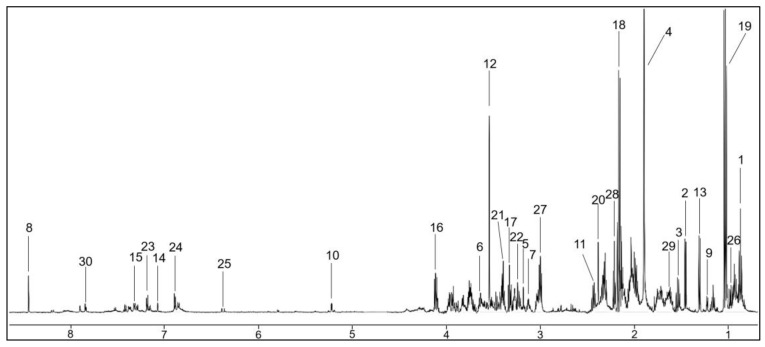
A representative ^1^H-NMR spectrum of an NS-C saliva sample with identified metabolites. Identified peaks indicate the following metabolites: 1,3: Butyrate; 2: Alanine; 4: Acetate; 5: Choline; 6: Ethanol; 7: Ethanolamine; 8: Formate; 9: Fucose; 10: Glucose; 11: Glutamine; 12: Gly-cine; 13: Lactate; 14: Histidine; 15: Phenylalanine; 16,17: Proline; 18,19: Propionate; 20: Succinate; 21,22: Taurine; 23,24: Tyrosine; 25,30: Urocanate; 26: Valine; 27,28,29: 5-APN. HC, healthy control; 5-APN, 5-Aminopentanoate. NS-C, non-Sjögren’s control group.

Using nuclear magnetic resonance (NMR) spectroscopy analysis, each processed saliva sample generated a ^1^H-NMR spectrum that was further analyzed by Chenomx software (Version 9.0) (Figure 2). Thirty-four metabolites were identified and quantified and were consistent, in part, with previously published reports (Supplementary Table S2).

**Figure 3:**
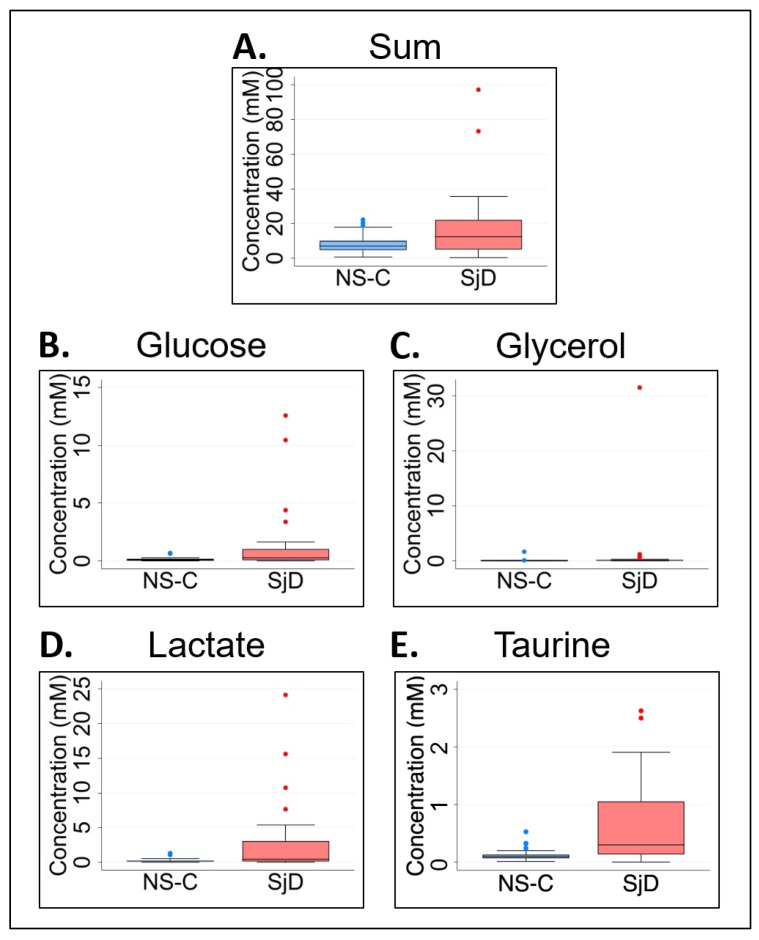
Metabolite concentration analysis of saliva samples from pSjD and NS-C subjects. No significant difference was found between the sum metabolite concentrations of saliva samples from the NS-C and the SjD groups (**A**). The median concentration levels of glucose, glycerol, lactate, and taurine (**B**–**E**) were significantly higher in saliva samples from pSjD subjects in comparison to NS-C. The blue and red dots above the box plots represent outlier data points outside of the interquartile range. NS-C, non-Sjögren’s control, blue box plots; SjD, Sjögren’s syndrome, red box plots.

We analyzed non-normalized metabolite concentrations in saliva samples as a practical and diagnostic approach that can be performed in the clinical field. Because sample concentration normalization (as a percentage of the sum of observed concentrations) is a well-recognized and commonly practiced analysis in metabolomics [51], we also analyzed normalized metabolite data to compare the samples from the pSjD and NS-C groups (Table 1). First, non-normalized data (metabolite concentrations in mM values) were compared between the pSjD and NS-C groups. Although significant association was found between subjects’ sex and group (*p* < 0.02, Figure 1A), and median age and group (*p* < 0.001, Figure 1B), there was no significant difference in the median sum of salivary metabolite concentrations from the pSjD and NS-C groups (*p* = 0.114, Figure 3A). In analyzing non-normalized data and accounting for false discovery rate (FDR) using Benjamini–Hochberg correction, significant differences in the median levels of glucose (*p* = 0.0442), glycerol (*p* = 0.051), lactate (*p* = 0.0272), and taurine (*p* < 0.068) were identified between the two study groups (Table 1).

In comparison to the NS-C group, the concentration of these four metabolites was elevated in the pSjD group (Figure 3B–E). However, we found that prior to *p* value FDR correction, six additional metabolites—choline, dimethylamine, acetoin, fructose, pyruvate, and valine—were significantly higher in saliva samples from pSjD subjects in comparison to the NS-C group (Supplementary Figure S2 and Supplementary Table S3).

**Figure 4:**
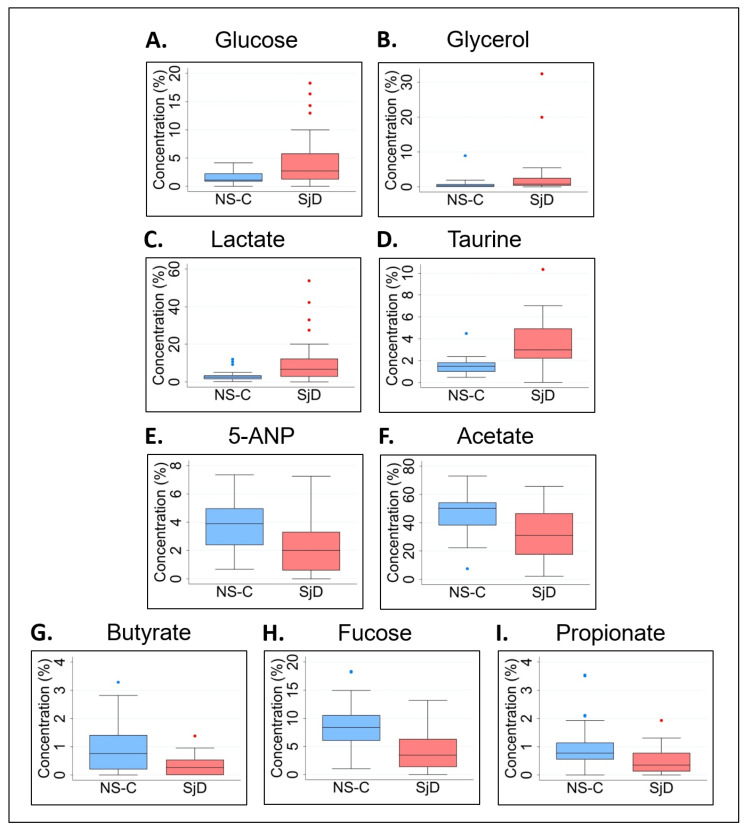
Metabolite normalized data analysis of saliva samples from NS-C and pSjD subjects. The median normalized concentration levels of glucose, glycerol, lactate, and taurine (**A**–**D**) were significantly higher, whereas the median normalized concentration levels of 5-ANP, acetate, butyrate, fucose, and propionate (**E**–**I**) were significantly lower in saliva samples from pSjD subjects in comparison to NS-C controls. The blue and red dots above or below the box plots represent outlier data points outside of the interquartile range. NS-C, non-Sjögren’s control (blue box plots); SjD, Sjögren’s syndrome (red box plots); 5-ANP, 5-aminopentanoate.

Thereafter, analysis of normalized data while applying the same FDR correction revealed significant differences in median levels of glucose (*p* = 0.0289), glycerol (*p* = 0.0302), lactate (*p* = 0.0074), taurine (*p* = 0.0001), 5-aminopentanoate (5-ANP) (*p* = 0.0027), acetate (*p* = 0.0043), butyrate (*p* = 0.0048), fucose (*p* = 0.0117), and propionate (*p* = 0.0027) between the pSjD and NS-C groups (Table 1). In comparison to the NS-C group, the normalized median levels of glucose, glycerol, lactate and taurine were elevated in the pSjD group (Figure 4A–D), while 5-ANP, acetate, butyrate, fucose and propionate were lower in the pSjD group (Figure 4E–I). Prior to *p* value correction, two additional metabolites—choline and dimethylamine—were found to be significantly higher in saliva samples from the pSjD group in comparison to the NS-C group (Supplementary Figure S3 and Supplementary Table S3). Consistently, metabolite concentration and normalized data analyses indicated an increase in the levels of glucose, glycerol, lactate and taurine in saliva samples from pSjD subjects when compared to NS-C subjects (Figure 3B–E and Figure 4A–D).

Table 1 also presents the fold change (FC) and percent change in the median levels of glucose, glycerol, lactate, taurine, 5-ANP, acetate, butyrate, fucose, and propionate in both metabolite concentration and normalized data analyses. While the values varied between the metabolite concentration data and normalized data analyses, the trends of elevated or reduced levels of these metabolites in saliva samples from the pSjD group compared to the NS-C group remained the same (Table 1).

For both analyses, the increase in the percent change of metabolites that were found to be significantly elevated in the pSjD group varied between 177% (glucose) and 200% (taurine) for metabolite concentration, and between 98% (glycerol) and 172% (lactate) for the normalized data. Metabolites that were significantly higher in NS-C relative to pSjD in the normalized data analysis only varied between 62% (acetate) and 184% (butyrate).

Using normalized data, the mean concentration values of metabolites that showed significant elevated or reduced levels in saliva samples from the pSjD group in comparison to the NS-C group were analyzed with a volcano plot (Supplementary Figure S4). In agreement with the data in Table 1, saliva samples from pSjD patients had higher levels of taurine, lactate, and glycerol, and lower levels of propionate, butyrate, 5-ANP, acetate and fucose, in comparison to samples from the NS-C group.

**Figure 5:**
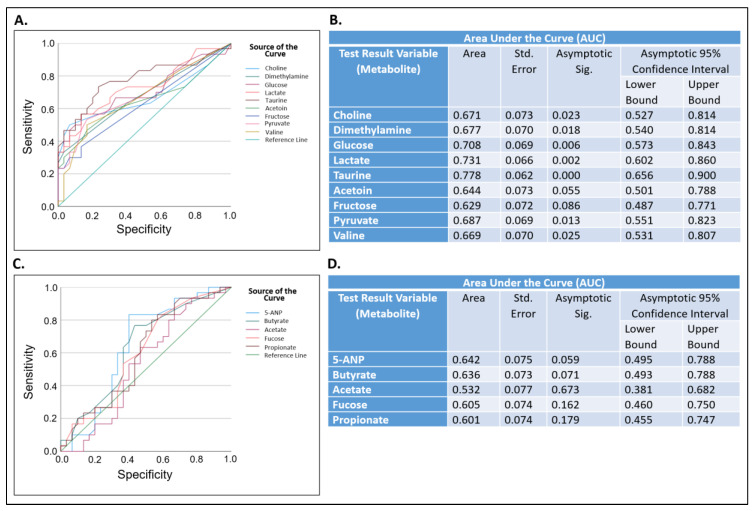
Significantly higher or lower metabolite levels as potential biomarkers for primary Sjögren’s disease prediction. Receiver operating characteristic (ROC) curve analysis (**A**,**C**) and AUC analysis (**B**,**D**) for specific metabolites that were significantly elevated (**A**,**B**) or lower (**C**,**D**) in saliva samples from the primary Sjögren’s disease group in comparison to the non-Sjögren’s control group. AUC, area under the curve; Std. Error, standard error; Asymptotic Sig., *p* value; 5-ANP, 5-aminopentanoate.

Next, receiver operating characteristic (ROC) analysis were performed. This was conducted to determine if metabolites that were significantly altered in their concentration in samples from the pSjD group (Table 1) may serve as potential biomarkers that can distinguish between saliva samples from pSjD subjects and from those who are not affected by Sjögren’s disease (Figure 5).

ROC analysis is useful in assessing the performance of diagnostic tests and, more generally, the accuracy of a statistical model that classifies subjects into one of two categories, such as “diseased” or “not diseased” [52]. In that analysis, an area under the curve (AUC) between 0.70–0.80 is considered acceptable, between 0.80–0.90 is considered excellent, and >0.90 is considered outstanding [53]. We generated ROC curves for metabolites that were significantly higher (Figure 5A) or lower (Figure 5C) in their levels in saliva samples from the pSjD group in comparison to the NS-C group (Table 1). For complete analysis, metabolites that showed significant *p* values before applying the Benjamini–Hochberg correction for FDR were also included (Supplementary Table S3). The AUCs of taurine (0.78), lactate (0.73), and glucose (0.71) were greater than 0.70 with significant *p*-values (*p* < 0.001, *p* = 0.002 and *p* = 0.006, respectively) (Figure 5A,B). The AUCs for choline, dimethylamine, pyruvate, and valine were lower than 0.70 with significant *p* values, and the AUCs of acetoin and fructose were greater than 0.60 with *p*-values that were not significant (Figure 5B). The AUCs of 5-ANP, butyrate, acetate, fucose, and propionate were lower than 0.70 with *p*-values that were not significant (Figure 5C,D).

### 2.3. The Effect of Age on Salivary Metabolic Profiles

Since Sjögren’s disease is mostly age dependent [5,6], we also analyzed a subset of the data that was obtained in this study in which the saliva samples were from pSjD and NS-C subjects between the ages of 30–70 years old. This age-restricted subset was comprised of 38 samples, of which 24 were from the pSjD group and 14 were from the NS-C group (Supplementary Figure S5). The pSjD group included twenty-two females (91.7%) and two males (8.3%), whereas the NS-C group included eleven females (78.6%) and three males (21.4%) (Supplementary Figure S5A,C). There was no significant association between sex and subject group (*p* = 0.34), and no significant difference in the median age of these sub-groups (*p* = 0.08). The median ages were 60.5 (iqr = 10.5) in the pSjD group and 49.5 (iqr = 28) in the NS-C group (Supplementary Figure S5B,C). Nonetheless, in analyzing metabolite concentrations and accounting for FDR using Benjamini–Hochberg correction, the level of glycerol only (*p* = 0.0102) was elevated in saliva samples from the age-restricted pSjD sub-group in comparison to the NS-C sub-group (Table 2). However, prior to *p* value correction, a significant increase in glucose, taurine, fructose, and lactate, as well as in choline (borderline significance), and a significant decrease in 5-ANP, butyrate, and propionate were revealed in saliva samples from the age-restricted pSjD sub-group compared to the NS-C sub-group (Supplementary Figure S6 and Supplementary Table S3).

**Figure 6:**
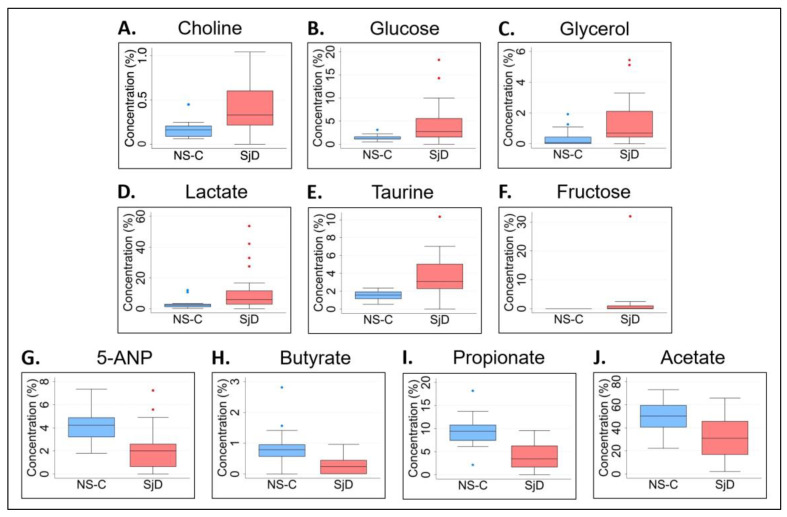
Metabolite normalized data analysis of saliva samples from a subset of age-restricted, pSjD and NS-C study participants between 30 and 70 years old. The median normalized concentration levels of choline, glucose, glycerol, lactate, taurine, and fructose (**A**–**F**) were significantly higher, while the median normalized concentration levels of 5-ANP, butyrate, propionate, and acetate (**G**–**J**) were significantly lower, in saliva samples from age-restricted pSjD subjects in comparison to NS-C controls. The blue and red dots above and below the box plots represent outlier data points outside of the interquartile range. NS-C, non-Sjögren’s control group (blue box plots); SjD, Sjögren’s disease group (red box plots); 5-ANP, 5-aminopentanoate.

In contrast to metabolite concentration data, normalized data analysis with corrected *p* values for FDR indicated significantly higher levels of choline (*p* = 0.0191), glucose (*p* = 0.0194), taurine (*p* = 0.0041), and glycerol (*p* = 0.0148), and borderline significance of lactate and fructose levels (*p* = 0.0559 and *p* = 0.0544, respectively), in saliva samples from the age-restricted pSjD sub-group in comparison to the NS-C sub-group (Figure 6A–F and Table 2). Additionally, this analysis revealed significantly lower levels of 5-ANP (*p* = 0.0034), butyrate (0.0041), propionate (*p* = 0.0003), and acetate (*p* = 0.0148) in the pSjD group in comparison to the NS-C group (Figure 6G–J and Table 2). Prior to *p* value correction, the levels of these metabolites were found to be significant between saliva samples from the age-restricted SjD and NS-C sub-groups, as well (Supplementary Table S3).

The fold change (FC) and percent change in the median levels of these metabolites in both non-normalized (metabolite concentration) and normalized data analyses are also presented in Table 2. For both analyses, the increase in percent change of metabolites that were found to be significantly elevated in saliva samples from this age-restricted subset of the pSjD group relative to the NS-C group varied between 138% (choline) and 1033% (glycerol) for metabolite concentration, and between 96% (taurine) and 659% (glycerol) for the normalized data (Table 2). Conversely, the percent change of metabolites that were significantly lower in samples from this age-restricted subset of the NS-C group relative to the pSjD group varied between 163% (5-ANP) and 508% (butyrate) for metabolite concentration, and between 63% (acetate) and 222% (butyrate) for the normalized data (Table 2). In agreement with these results, using the normalized age-restricted subset of data, a volcano plot shows that the level of five metabolites (taurine, choline, glucose, glycerol and lactate) were higher, whereas four metabolites (propionate, 5-ANP, acetate, and butyrate) were lower in saliva samples from age-restricted subset of pSjD patients in comparison to samples from non-Sjögren’s controls (Supplementary Figure S7).

**Figure 7:**
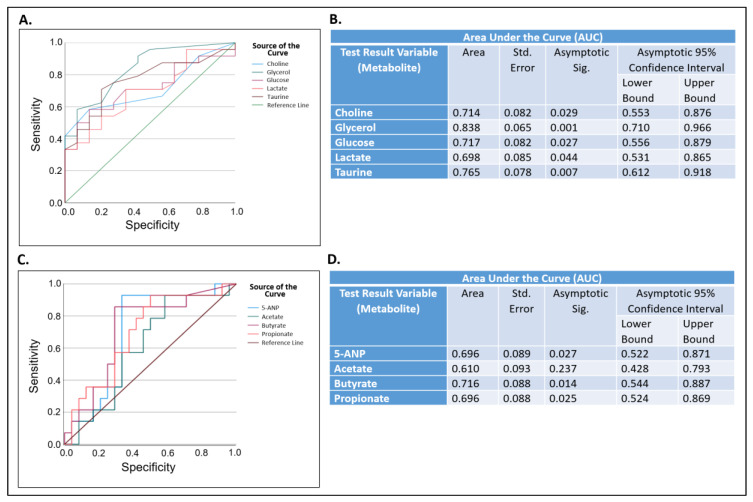
Significantly higher or lower metabolite levels as potential biomarkers for primary Sjögren’s disease prediction in 30-70 years old subjects. Receiver operating characteristic (ROC) curve analysis (**A**,**C**) and AUC analysis (**B**,**D**) for specific metabolites that were significantly elevated (**A**,**B**) or lower (**C**,**D**) in saliva samples from a subset of 30-70 years old subjects in the primary Sjögren’s disease group in comparison to the non-Sjögren’s control group. AUC, area under the curve; Std. Error, standard error; Asymptotic Sig., *p* value; 5-ANP, 5-aminopentanoate.

Next, we performed ROC curve analysis of these nine metabolites. Of the five metabolites that were significantly higher, the AUC of glycerol (0.84) was greater than 0.80, the AUCs of choline (0.71), glucose (0.72), and taurine (0.77) were greater than 0.70, and the AUC of lactate (0.698) was borderline at 0.70 with significant *p* values (*p* = 0.001, *p* = 0.029, *p* = 0.027, *p* = 0.007, and *p* = 0.044, respectively) (Figure 7A,B). Of the four metabolites that were significantly lower, the AUC of butyrate (0.716) was greater than 0.7 while 5-ANP and propionate (0.696) were borderline at 0.70, with significant *p* values (*p* = 0.014, *p* = 0.027, and *p* = 0.025, respectively). Only the AUC of acetate was lower than 0.70 with no significant difference (AUC = 0.610, *p* = 0.237) (Figure 7C,D).

## 3. Discussion

Salivary metabolomics has been implemented in the study of different diseases, and contributed to the discovery of varied, disease-specific biomarkers. Here, we present nuclear magnetic resonance (NMR) spectroscopy-based metabolomics of whole, unstimulated saliva from 30 participants with primary Sjögren’s disease (pSjD) in comparison to 30 participants who do not have Sjögren’s disease (non-Sjögren’s control group, NS-C). Our findings suggest that NMR-based salivary metabolomics can differentiate between pSjD patients and NS-C group, and stress the importance of participant age range selection, inclusion of both sexes from diverse race/ethnicity, and the use of both non-normalized and normalized data analysis in defining metabolic signatures of pSjD patients.

Epidemiologic studies that investigated the prevalence and incidence of Sjögren’s syndrome emphasized that this disease affects individuals of all races and ethnicities [1,2,3,4,54], and indicates that pSjD is more prevalent in Caucasian women [1,2,3]. Additionally, pSjD has a higher frequency in females than in males [1,2,3]. Using data extracted from the Manhattan Lupus Surveillance Program, a population-based retrospective registry of Systemic Lupus Erythematosus and related diseases in a diverse population, Izmirly et al., evaluated the prevalence and incidence rate of pSjD among residents in Manhattan, NY, between 2007 to 2009. The study revealed significant differences in the incidence and prevalence of pSjD between females and males, and significant differences in the incidence of pSjD by race/ethnicity among women, with the highest rate amongst Caucasian and Asians [54]. The authors indicated the scarcity of studies on pSjD among multi-racial/ethnic populations. Concurring with the importance of inclusion of diverse patient populations in research studies, our pilot study was open to the enrollment of subjects from all races/ethnicities with the expectation that participants reflect the population of the Greater Boston area. It represented a multi-racial/ethnic population, and while over 70% of study participants were White, 13% were Asian, 5% were African American, 3% were American Indian/Alaska Native, 2% reported more than one race and 5% did not report their race. Additionally, in contrast to multiple metabolomics studies that analyzed saliva samples from pSjD female patients only [43,44,45,47,48] and similar to a recent study by Li et al. [46], both the pSjD and healthy control groups in our study included females and males.

Epidemiologic studies also indicate that although pSjD is most common in middle-aged women, typically between 50–60 years old, the disease also affects men and can present at any age [4,5,6]. Therefore, our study was open to the enrollment of pSjD patients and NS-C participants at the ages between 18–80 years. This wide range of subject’s age encompassed the ages of participants in other salivary metabolomics studies of pSjD. However, while our study included saliva samples from 30 pSjD subjects and 30 NS-C, these two groups were not age- and sex-matched. The data showed a significant difference (*p* < 0.001) in the median age of the pSjD group (63, iqr = 14) and the NS-C group (29 years, iqr = 21), and subjects’ sex and groups were significantly associated (*p* < 0.02). Nonetheless, there was no significant difference in the median sum of metabolite concentrations in saliva samples from the two study groups (*p* = 0.114), which was fundamental for conducting the non-normalized and normalized data analysis. The wide range of age was narrowed down in the age-restricted (30–70 years) subset of data, which included saliva samples from 24 pSjD subjects and 14 NS-C participants. In that subset, there was no significant difference (*p* = 0.08) in the median age between of the pSjD group and the NS-C group, and there was no significant association between sex and subject group (*p* = 0.34). In our study, the mean age of the complete pSjD group (*n* = 30) and the NS-C group (*n* = 30) was 62.2 ± 10.2 and 37.2 ± 15.6. In the age-restricted subset of data, the mean age of the set the pSjD group (*n* = 24) and the NS-C group (*n* = 14) was 59.3 ± 9.1 and 50.4 (±13.7). To our knowledge, only a handful of articles have been reported on salivary metabolomics of pSjD using either NMR or mass spectrometry (MS) techniques. The mean age of study participants ranged from 48.6 to 65.4 years in studies that used NMR technology [43,44,48], and from 39.7 to 53.2 years in studies that used MS technology [45,46,47]. These studies included females only and the pSjD group varied between 7 to 19 participants. Using MS technology, the study by Li et al. included both age- and sex-matched females and males with a mean age of 39.7 and 40.6, respectively, and the pSjD group was composed of 32 participants [46], which was similar to the number of pSjD participants in our study.

In this study, we analyzed both non-normalized (metabolite concentration) and normalized (metabolite normalization) data of saliva samples from the entire study population (*n* = 60) and in a subset of age-restricted participants who were between 30–70 years old (*n* = 38) from both study groups. Using data normalization per total metabolite abundance in each sample reduced the variation between the expression levels of individual salivary metabolites in each of the samples from both study groups. Using NMR, we identified 34 metabolites in saliva samples from pSjD and NS-C study participants. While accounting for the false discovery rate (FDR), metabolite concentration and normalized data analyses of the complete study population, as well as normalized data of the age-restricted subset of participants (30–70 years), revealed a significant increase in the levels of glucose, glycerol, taurine, and lactate, in saliva samples from the pSjD group in comparison to their levels in the NS-C group. Additionally, normalized data of saliva samples from both the complete study population and the age-restricted subset revealed a significant decrease in several related carboxylic acids (5-aminopentanoate (5-ANP), acetate, butyrate, and propionate) in pSjD subjects compared to the controls. Moreover, compared to the NS-C group, normalized data of saliva samples from the pSjD group showed a significant increase in choline in the age-restricted subset data, and a significant decrease in fucose in the whole study population. We conducted receiver operating characteristic (ROC) analysis of the metabolites that showed a significant increase or decrease in their levels (with and without false discovery rate correction) of the complete study population as well as the age-restricted subset of study participants (30–70 years old). This analysis revealed that from the metabolites that were significantly increased in the complete pSjD group (*n* = 30), the area under the curve (AUC) of taurine, lactate and glucose, were greater than 0.70, and in the age-restricted subset of this group, the AUC of glycerol, taurine, choline, and glucose were greater than 0.70, while lactate was borderline at 0.70. From the metabolites that were significantly lower in the pSjD group, the AUC of butyrate was greater than 0.70, and the AUC of 5-ANP propionate were borderline at 0.70. These results suggest that taurine, lactate, glucose, glycerol, choline, butyrate, and potentially propionate and 5-ANP, as well as combinations between these metabolites, might serve as biomarkers that differentiate between pSjD patients and non-Sjögren’s subjects.

The biological functions of taurine, an abundant free amino acid in humans, include antioxidant activity, osmoregulation, membrane stability, maintenance of calcium concentrations and homeostasis of cells. High concentrations of taurine are detected in tissues exposed to chronic inflammatory and oxidative stress [55]. Choline, a quaternary amine, serves as a component of structural lipoproteins, as well as blood and membrane lipids. Choline-containing metabolites are components of the phospholipid metabolism of cell membranes, and are linked to breast, brain, and prostate cancers [56]. Moreover, choline is associated with retinal development and differentiation, and being a precursor of the neurotransmitter acetylcholine, it is involved in the production and secretion of tears by lacrimal glands. Choline deficiency is linked to multiple retinal disorders, including dry eye syndrome [57]. Both taurine and choline were found to be elevated in the saliva of patients with oral squamous cell carcinoma, and have been suggested as potential biomarkers for this disease [56,58].

The results of our study only partially align with previous NMR-based and MS-based salivary metabolomics studies in pSjD patients in comparison to the NS-C group of participants. For example, the significant increase in the level of taurine in pSjD patients in our study is in agreement with the findings of the studies by Mikkonen et al. [43] and of Harella et al. [44]. Conversely, the study by Hynne et al. [47] found no significant differences in the levels of taurine in pSjD and NS-C study subjects. Additionally, the significant decrease in the level of fucose in the normalized salivary metabolomics data of the pSjD group in comparison to the NS-C group in our study is in agreement with the findings of Kageyama et al. [45] and Setti et al. [48] studies. Notably, while a significant decrease in butyrate level in pSjD was identified in the normalized data of all study participants and the analyses of the age-restricted subset of data in our study, butyrate level was significantly increased in pSjD patients in the studies by Mikkonen et al. [43] and of Setti et al. [48]. Among the 34 metabolites that were analyzed in our study, we found no significant differences in the levels of alanine, glycine, phenylalanine, proline, tyrosine, aspartic acid, and uric acid between the pSjD and NS-C study groups. In contrast, the study by Mikkonen et al. [43] demonstrated a significant increase in alanine, glycine, phenylalanine and proline, whereas Li et al. [46] showed an increase in tyrosine, phenylalanine, proline and aspartic acid in pSjD patients. Conversely, Kageyama et al. [45] reported a significant decrease in glycine, tyrosine and uric acid, and Setti et al. [48] indicated a decrease in glycine, tyrosine and proline in pSjD patients in comparison to NS-C individuals.

The similarities and discrepancies between the metabolic profiles of pSjD patients and NS-C participants in our study and the aforementioned studies can be attributed, at least in part, not only to participant-related factors, such as sex, age and race/ethnicity, but also to differences in study settings and implementation of the false discovery rate in the statistical analysis of the data, as well as the methodology of saliva collection and the analytical techniques used. The main analytical techniques employed in salivary metabolomics of pSjD are NMR spectroscopy and mass spectrometry (MS) that provide data on the relative and total concentrations of different classes of metabolites in a single measurement. The analytical advantages of ^1^H-NMR over MS include high reproducibility; small sample volume (100–500 μL); minimal sample processing prior to the analysis; and an unbiased analysis of a subset of known compounds as well as the whole array of metabolites within the NMR detection limit (about 1 μM), which allows the measurement of relatively low-abundance compounds within the sample [49,50]. A limitation of using HPLC-MS in the global metabolism is that the majority of the detected metabolites are not identified with their unique name and function since only a limited number of known metabolites have defined experimental spectral data in databases. Multiple sampling protocols have been established and compared in the collection and analysis of both whole unstimulated and stimulated saliva samples to study salivary flow rate and content, saliva function and dysfunction and microbial diversity of the oral cavity ecosystem, in health and disease [59,60]. Considered as a ‘gold-standard’, unstimulated saliva provides a consistent oral fluid sample, which reflects the contribution of the major and minor salivary glands, whereas stimulated saliva provides a larger sample volume that reflects localized secretion from specific salivary glands [61]. Unstimulated saliva can be collected by passive drooling, and stimulated saliva can be collected following gustatory stimulation or mastication by chewing on a sterile paraffin wax.

In our study, NMR-based metabolomics was implemented in the analysis of unstimulated saliva samples from pSjD patients and NS-C subjects. In their studies with pSjD patients, Setti et al. analyzed unstimulated saliva samples [48], while Mikkonen et al. [43] and Herrala et al. [44] analyzed stimulated saliva samples by ^1^H-NMR spectroscopy. Kageyama et al. [45] and Li et al. [46] analyzed unstimulated saliva samples with gas chromatography-mass spectrometry (GC-MS) and ultra-performance liquid chromatography-high-resolution mass spectrometry (UPLC-HRMS), respectively, while Hynne et al. analyzed stimulated saliva samples with high performance liquid chromatography-high resolution mass spectrometry (HPLC-MS) [47]. Using either NMR, MS, or both analytical techniques, several studies indicated differences in salivary metabolic profiles of unstimulated and stimulated saliva samples. Using mass spectrometry-based salivary metabolomics, Maruyama et al. compared metabolic profiles of stimulated saliva, unstimulated saliva, and mouth-rinsed water from healthy subjects [62]. While 153 common metabolites were analyzed with these sampling methods, some metabolites were detected in only one or two of them and were not detected in the others [62]. Figueira et al. reported findings from a comparative metabolomics of unstimulated, stimulated, and parotid gland saliva using both targeted NMR spectroscopy and liquid chromatography-mass spectrometry (LC-MS) [63]. Although detected metabolites were almost identical, there were differences in the metabolite composition ratios between the three methods of saliva sample collection [63]. Using ^1^H-NMR technology, Takeda et al. [64] showed higher concentrations of almost all metabolites in unstimulated versus stimulated saliva samples in a cohort of healthy males, as well as higher concentrations of multiple metabolites in saliva samples from males as compared to females [64]. Okuma et al. used capillary electrophoresis mass spectrometry (CE-MS) to determine the quantitative relationship between masticatory induced stimulation and metabolite composition of saliva [65]. The study showed that the concentration of most of the metabolites in stimulated saliva was significantly higher, or almost constant, compared with those in unstimulated saliva. Additionally, the overall concentration of metabolites in these two sample types were associated with age and sex [65]. Therefore, further comparisons between the metabolic profiles of unstimulated and stimulated saliva samples from pSjD patients and those who are not affected by Sjögren’s disease may reveal complementary metabolite information, and should be considered in future studies.

This research study was a pilot study, and its inherent limitations should be recognized. Because we intended to conduct an inclusive survey of the population, without the limiting of age or gender, the study included saliva samples from 30 pSjD participants and 30 NS-C participants, which therefore were not age-matched. This resulted in significant differences in the median age of the two groups and a significant association between subjects’ sex and groups; it was accounted for in the age-restricted (30–70 years) subset of data. Nonetheless, participant age and sex matching between study groups should be considered in future studies. Based on the inclusion and exclusion criteria used in this study, study participants in the NS-C group did not have a history of any type of autoimmune disease, such as Sjögren’s or rheumatoid arthritis. Study subjects in the pSjD had a controlled condition without any features contraindicative to participation. Yet, known associated comorbidities with pSjD, such as acid reflux [66], can contribute to alterations in salivary metabolic profiles of pSjD patients. For example, Kageyama et al. showed that pSS patients with a history of major salivary glanditis, including enlarged major salivary glands, presented a different metabolic profile than pSjD patients without this condition [45]. With a larger cohort of subjects, correlations between salivary metabolic profiles, comorbidities and the use of different types of medications (i.e., sialagogues) could be investigated in future studies. Finally, while the use of ^1^H-NMR spectroscopy focused here on soluble, hydrophilic metabolites in saliva samples from study participants, the profiles of bioactive lipids and other hydrophobic metabolites in these samples were not measured. The composition and quantity of water soluble and non-soluble lipids and other compounds in saliva samples from pSjD patients may provide a more comprehensive representation of disease-specific salivary biomarkers for primary Sjögren’s disease.

## 4. Materials and Methods

### 4.1. Study Participants

Tufts Health Sciences Institutional Review Board (IRB) approved the protocols, materials and procedures of the study. This single-center, cross-sectional pilot research study is part of a larger collaborative research project between Tufts University School of Dental Medicine and School of Medicine (Boston, MA, USA). The study was conducted at the Oral Medicine clinic of Tufts University School of Dental Medicine and included one visit during the morning hours (09:00 am–12:00 pm) appointments to account for circadian rhythmicity that has been shown to control physiological phenomena, including salivary flow and content, during a 24 h day [67,68,69]. Individuals with primary Sjögren’s Disease (pSjD), and those who do not have Sjögren’s Disease (non-Sjögren’s control group, NS-C) were recruited through advertisements and referrals. Subjects who responded to recruiting contacts underwent an initial screening by telephone and were invited to participate if they met the inclusion criteria. Each study participant voluntarily signed a written informed consent form, and demonstrated the ability to understand the study purpose and comply with the study protocol. The study was open to both females and males, ages 18–80 years old. Subjects with pSjD met the revised American-European Consensus Group criteria for classification of primary Sjögren’s syndrome [11]. Individuals with pSjD had a controlled condition without any features contraindicative to participation; they were excluded from participation if they were affected by narcolepsy, Alzheimer’s disease or other diagnosed dementia, a psychotic disorder, and if they were undergoing chemotherapy or use of any investigational drugs or participation in a clinical trial within 3 months of the study visit. In addition to the exclusion criteria used for the pSjD group, individuals were excluded from the NS-C group if they had a known history of any type of autoimmune disease (e.g., Sjögren’s, rheumatoid arthritis, and systemic lupus erythematosus).

### 4.2. Data Collection

Sixty individuals were enrolled in the study, of which 30 were in the pSjD group and 30 were in the NS-C group. Study participants were asked to refrain from eating, drinking or smoking for 90 min prior to their study visit. At the study visit, participants underwent a standardized evaluation of the oral cavity, soft and hard tissues, following standard of care procedures in US dentistry, by a team of experienced dentists and dental specialists in order to determine potential pathological conditions. Demographic data including age, sex, race, medical history and existing features of oral dryness, presence of systemic metabolic alterations such as dyslipidemia and diabetes, presence of autoimmune diseases and a list of current medications were collected using a standardized Medical History form at Tufts University School of Dental Medicine.

### 4.3. Collection and Processing of Saliva Samples

Immediately before saliva sample collection, study participants rinsed their mouth with water. A whole, unstimulated saliva sample was collected from each subject using a saliva collection aid (Salimetrics, Carlsbad, CA, USA) that was placed in a 1.8 mL microfuge tube on ice. All saliva samples were stored at −20 °C prior to processing, similar to previous studies [43,44]. Samples were thawed on ice and centrifuged at 1800× *g* for two minutes at 4 °C, and 350 μL from each supernatant were transferred to a fresh Eppendorf tube. The supernatant volume was determined by pipetting and, if necessary, was increased to 300 μL using de-ionized water. Dilution factors were taken into account when final metabolite concentrations were determined. Samples were centrifuged at 9000× *g* for five minutes at 4 °C, and 300 μL from each supernatant was transferred into a fresh tube. For each sample, 200 μL of de-ionized water and 56 μL of buffer (500 mM phosphate, 5 mM sodium trimethylsilylpropanesulfonate (DSS) in deuterium oxide (D_2_O), 0.02% sodium azide) were added, and samples were vortexed for 15 s. Finally, 500 μL from each sample was transferred into Wilmad nuclear magnetic resonance glass tubes (Vineland, NJ, USA).

### 4.4. Nuclear Magnetic Resonance Spectroscopy

Sequentially marked, de-identified processed saliva samples were subjected to nuclear magnetic resonance (NMR) spectroscopy analysis by an operator who was blinded to the source (pSjD or NS-C) of the samples. A ^1^H-NMR spectrum was recorded for each saliva sample on a Bruker Avance 600 spectrometer (Bruker Corporation, Billerica, MA, USA), at 25 °C, using a zgesgppe pulse sequence. A total of 128 scans were acquired into 76,922 data points, a spectral width of 16 ppm, 4 s acquisition time, and a relaxation delay of 1 s. The data were exported to Chenomx (Version 9.0, Chenomx Inc., Edmonton, AB, Canada) for further processing. NMR spectra were phase corrected and baseline corrected. The water region (4.5–5.0 ppm) was excluded from analysis. Salivary metabolites were identified using the chemical shift values and signal splitting patterns reported in the literature and the reference library within Chenomx. An internal standard of DSS (sodium trimethylsilylpropanesulfonate) was used for chemical shift referencing (δ 0.0). The area under the spectral line for each compound (the integral) is directly proportional to the concentration of each metabolite including the 0.5 mM DSS internal standard. Metabolite concentrations were exported to Excel for further analysis.

### 4.5. Statistical Analysis

To date, only a limited number of studies using NMR [43,44,48], or mass spectroscopy [42,45,46,47], techniques for saliva metabolomics in pSjD patients have been reported. Using ^1^H-NMR spectroscopy, Mikkonen et al. reported that saliva samples from pSjD patients showed significantly higher levels of metabolites, such as alanine, glycine, phenylalanine, and proline than in NS-C [43]. Based on the reported mean concentrations of these metabolites, a power calculation was made assuming a type I error rate of 5% and a type II error rate of 20% for each of these metabolites. To determine significance, a minimum sample size for alanine (*n* = 14), glycine (*n* = 15), phenylalanine (*n* = 21), and proline (*n* = 23) was required. To ensure a representative sample, the sample size was increased to 30 subjects in each of the pSjD and NS-C groups in this study, resulting in 90–99% power for these metabolites (alanine 99%; glycine 99%; phenylalanine 92%; proline 90%). Descriptive statistics were calculated, including counts and percentages for categorical demographic variables, means and standard deviations for continuous variables, and median and interquartile ranges for non-normally distributed continuous metabolite levels. Differences in categorical variables between the pSjD and NS-C groups were compared with either the chi-square test or Fisher’s exact test. Non-normalized (metabolite concentration) and normalized data (metabolite normalization) were analyzed. For non-normalized data, the concentration of individual metabolites in each of the saliva samples was analyzed. For normalized data, the concentrations of individual metabolites were divided by the sum of the concentration of all identified metabolites in each saliva sample. By doing so, normalized data showed the percentage that each individual metabolite comprises of the overall metabolite concentration in each sample. The distribution of the data was assessed graphically with quantile-quantile plots (q-q plots) and histograms. Since the data were not normally distributed, the non-parametric Mann–Whitney U test was used to compare median metabolite levels between pSjD and NS-C groups in non-normalized and normalized data. Box plots were created to visualize the spread of the data, including range, interquartile range, median, and any outliers. Stata 17 (Stata Corp LLC, College Station, TX, USA) was used for the analysis. Calculation of Volcano Plots were performed using normalized data in MetaboAnalyst 5.0. Receiver Operating Characteristic (ROC) curves were made by IBM SPSS Statistics for Windows (version 27, Armonk, NY, USA: IBM Corp). Results were considered statistically significant at *p* ≤ 0.05. *p* values were also adjusted to account for the false discovery rate using Benjamini–Hochberg (BH) correction.

## 5. Conclusions

Early diagnosis of Sjögren’s disease, which can lead to early treatment of symptomatic care for mucosal signs and dryness symptoms, is critical for decreasing the complications and comorbidities that are associated with the progression of this illness. The study showed that NMR-based metabolomics can define salivary metabolic profiles that are associated with pSjD. This approach has the potential to serve as a non-invasive tool that can be integrated in the dental clinic workflow, allow for differential diagnosis of pSjD patients, and monitor disease progression, all of which will lead to the improvement of patients’ oral and overall health.

## Figures and Tables

**Table 1:** Salivary metabolites that are significantly different in their levels between pSjD and NS-C groups.

Metabolite	Metabolite Concentration (mM)		*p* Value Corrected	FC	Normalized Data		*p* Value Corrected	FC
**Metabolites significantly higher in pSjD relatively to NS-C** **in both Metabolite Concentration and Normalized Data**								
	p50 (iqr)			FC (%)	p50 (iqr)			FC (%)
	NS-C	pSjD			NS-C	pSjD		
Glucose	0.100 (0.160)	0.277 (0.947)	0.0442	1.8 (177%)	1.158 (1.447)	2.760 (4.585)	0.0289	1.4 (138%)
Glycerol	0.027 (0.510)	0.071 (0.177)	0.0051	1.6 (163%)	0.389 (0.803)	0.770 (2.149)	0.0302	1.0 (98%)
Lactate	0.189 (0.162)	0.432 (2.910)	0.0272	1.3 (129%)	2.484 (1.984)	6.753 (9.361)	0.0074	1.7 (172%)
Taurine	0.100 (0.063)	0.300 (0.917)	0.0068	2.0 (200%)	1.481 (0.837)	2.971 (2.751)	0.0001	1.0 (101%)
**Metabolites significantly lower in pSjD relatively to NS-C** **in Normalized Data analysis only**								
5-ANP	0.218 (0.291)	0.123 (0.350)	0.1580	0.8 (77%)	3.893 (2.588)	2.013 (2.706)	0.0027	0.9 (93%)
Acetate	3.489 (3.678)	2.620 (6.843)	0.7328	0.3 (33%)	50.133 (16.004)	30.961 (29.140)	0.0043	0.6 (62%)
Butyrate	0.061 (0.075)	0.016 (0.094)	0.1544	2.8 (281%)	0.766 (1.210)	0.270 (0.544)	0.0048	1.8 (184%)
Fucose	0.060 (0.108)	0.039 (0.103)	0.2905	0.5 (54%)	0.782 (0.592)	0.356 (0.657)	0.0117	1.2 (120%)
Propionate	0.535 (1.170)	0.292 (0.940)	0.2833	0.8 (83%)	8.368 (4.524)	3.488 (4.980)	0.0027	1.4 (140%)

FC, fold change; pSjD, primary Sjögren’s disease; NS-C, non-Sjögren’s control, p50, median; iqr, interquartile range; 5-ANP, 5-aminopentanoate.

**Table 2:** Salivary metabolites that are significantly different in their levels in saliva samples from a subset of 30-70 years old subjects in the pSjD and NS-C groups.

Metabolite	Metabolite Concentration (mM)		*p* Value Corrected	FC	Normalized Data		*p* Value Corrected	FC
**Metabolites significantly higher in pSjD relatively to NS-C** **in Metabolite Normalized Data**								
	p50 (iqr)			FC (%)	p50 (iqr)			FC (%)
	NS-C	pSjD			NS-C	pSjD		
Choline	0.016 (0.012)	0.038 (0.083)	0.1927	1.4 (138%)	0.161 (0.121)	0.330 (0.392)	0.0191	1.0 (105%)
Glucose	0.096 (0.146)	0.277 (0.925)	0.2244	1.9 (189%)	1.158 (0.602)	2.768 (4.088)	0.0194	1.4 (139%)
Lactate	0.189 (0.294)	0.458 (2.508)	0.2129	1.4 (142%)	2.215 (1.965)	5.943 (8.914)	0.0559	1.7 (168%)
Taurine	0.099 (0.081)	0.299 (0.812)	0.0969	2.0 (202%)	1.578 (0.790)	3.093 (2.767)	0.0041	1.0 (96%)
*Fructose	0 (0)	0 (0.104)	0.1813		0 (0)	0 (1.119)	0.0544	
**Metabolites significantly higher in pSjD relatively to NS-C** **in both Metabolite Concentration and Normalized Data**								
Glycerol	0.006 (0.039)	0.068 (0.127)	0.0102	10.3 (1033%)	0.093 (0.450)	0.706 (1.694)	0.0148	6.6 (659%)
**Metabolites significantly lower in pSjD relatively to NS-C** **in Metabolite Concentration and Normalized Data**								
5-ANP	0.302 (0.426)	0.115 (0.365)	0.2411	1.6 (163%)	4.230 (1.694)	2.013 (1.971)	0.0034	1.1 (110%)
Acetate	4.303 (3.636)	2.309 (6.407)	0.4423	0.9 (86%)	50.335 (19.184)	30.961 (28.922)	0.0148	0.6 (63%)
Butyrate	0.079 (0.064)	0.013 (0.088)	0.1836	5.1 (508%)	0.789 (0.394)	0.245 (0.453)	0.0041	2.2 (222%)
Propionate	0.776 (1.463)	0.236 (0.917)	0.2411	2.3 (229%)	9.431 (3.452)	3.488 (4.660)	0.0003	1.7 (170%)

FC, fold change; pSjD, primary Sjögren’s disease; NS-C, non-Sjögren’s control; p50, median; iqr, interquartile range; 5-ANP, 5-aminopentanoate. * Although the median value of fructose was very low in metabolite concentration and normalized data analyses, the Mann-Whitney U test also accounts for the large spread of the data seen in the pSjD group. This resulted in a borderline significance *p* value for fructose.

## Data Availability

Additional data, beyond the data presented in this study and the Supplementary Materials, are available upon request from the corresponding authors. Based on ethical, legal, and technical considerations, some data may be restricted and not publicly available in order to preserve subject confidentiality.

## Supplementary Materials

The following supporting information can be downloaded at: https://www.mdpi.com/article/10.3390/molecules28155891/s1, Figure S1: Treemap charts of the distribution of medical conditions in the study population. Figure S2: Metabolite concentration comparisons (prior to *p* value correction) of saliva samples from pSjD and NS-C groups. Figure S3: Metabolite normalized data comparisons (prior to *p* value correction) of saliva samples from pSjD and NS-C groups. Figure S4: Volcano plot analysis of normalized data of saliva samples from pSjD and NS-C subjects. Figure S5: Distribution of sex and age in 30–70 year old study subjects. Figure S6: Metabolite concentration comparisons (prior to *p* value correction) of saliva samples from a subset of age-restricted, pSjD and NS-C study participants between 30 and 70 years old. Figure S7: Volcano plot analysis of normalized data of saliva samples from a subset of age-restricted, pSjD and NS-C study participants between 30 and 70 years old. Table S1: Demographics and clinical characteristics of the study population. Table S2: Quantification of all identified metabolites in saliva samples from the study population using concentration data and normalized data. Table S3: *p* values of the concentration data and normalized data of all identified metabolites in saliva samples from the study population, alongside adjusted *p* values using Benjamini–Hochberg correction (BH) to account for false discovery rate.
