# Supplementary material for: Identification of Salivary Metabolic Signatures Associated with Primary Sjögren’s Disease
Source: Molecules. 2023 Aug 5;28(15):5891. doi: 10.3390/molecules28155891 (PMC10421170; doi:10.3390/molecules28155891)
Supplement: Supplementary file 1 [file molecules-28-05891-s001.zip › molecules-2423968-supplementary.pdf]

A.

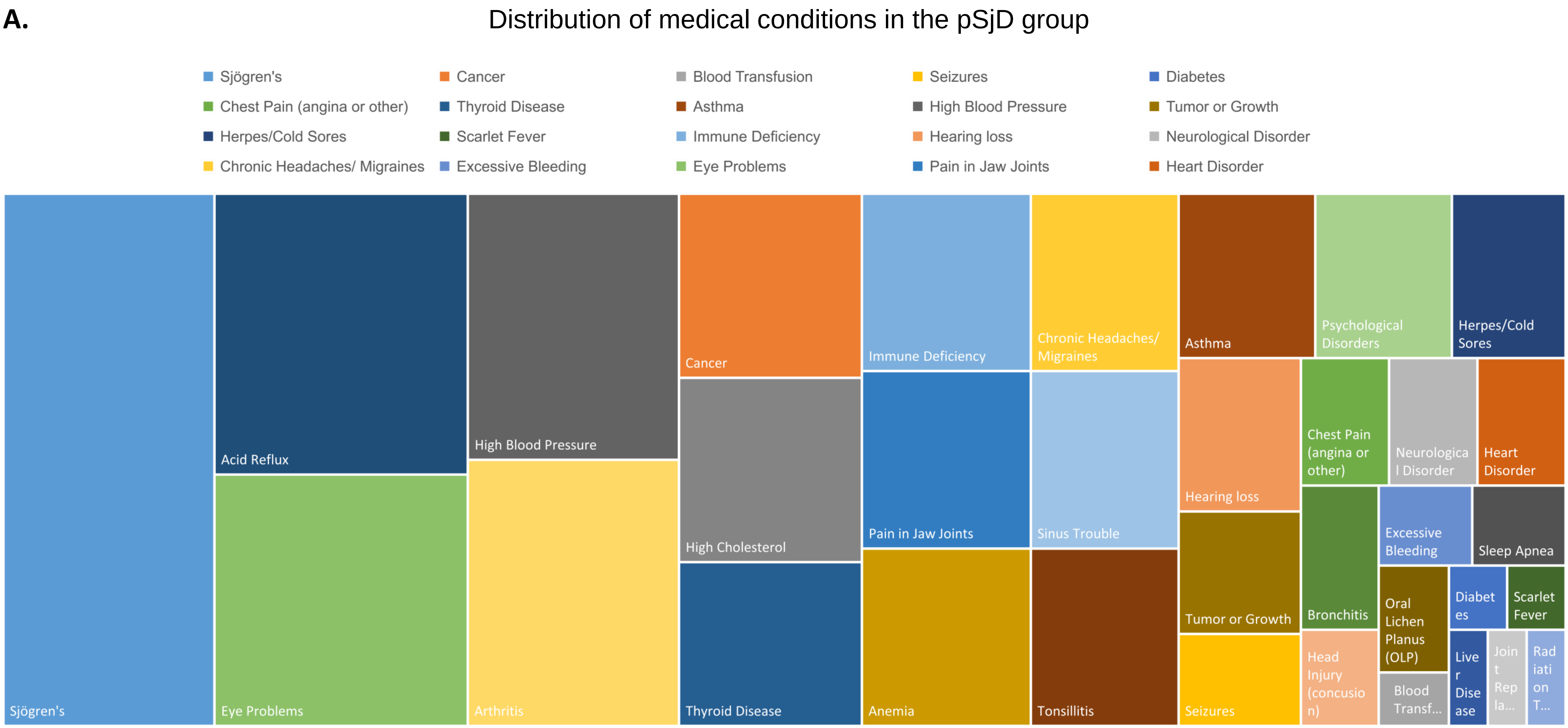

Supplementary Figure S1. Treemap charts of the distribution of medical conditions in the study population. Using a standardized Medical History form during the study visit, study participants reported their medical conditions. The treemaps of these medical conditions in the pSjD group (A) and the NS-C group (B), are proportional for the number of the reported disorders. The numerical data for these treemap charts are provided in Table 1. pSjD, Sjögren's syndrome group; NS-C, non-Sjögren's syndrome control group).

B.

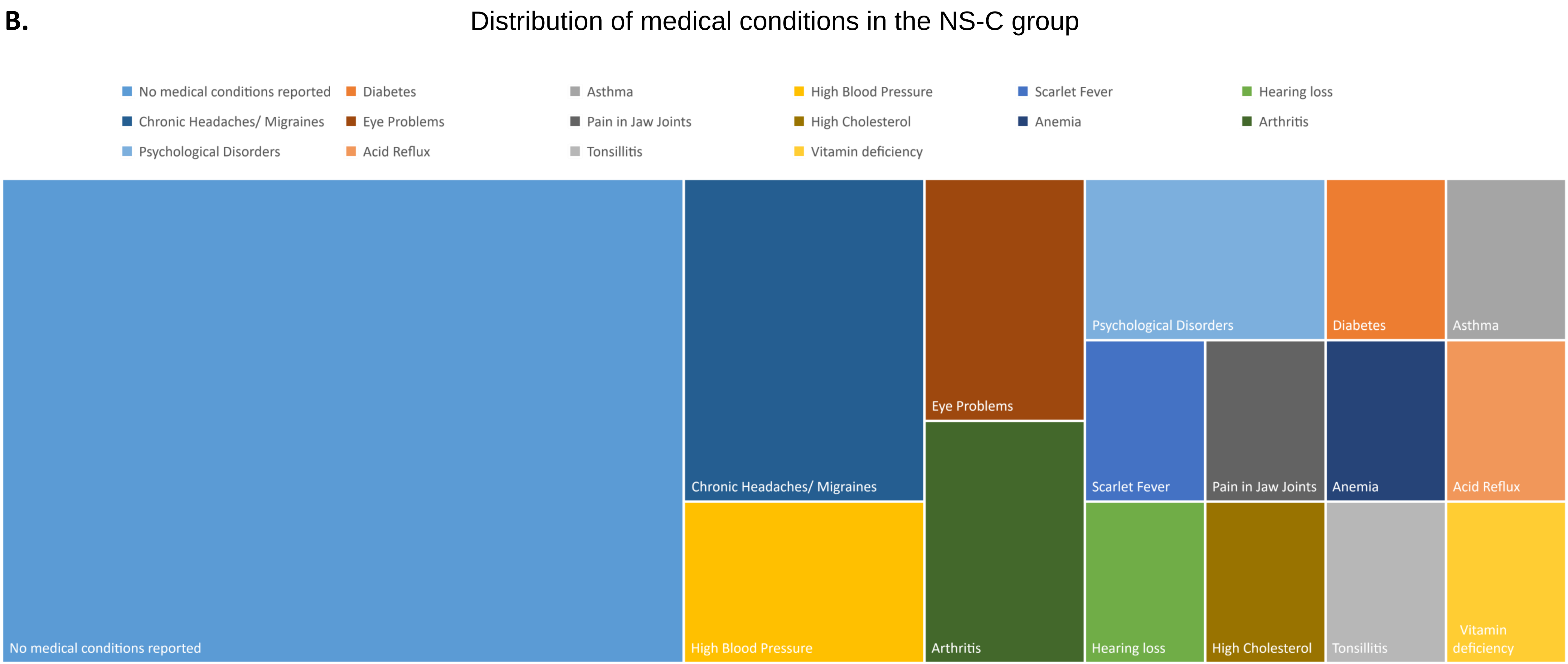

Supplementary Figure S1. Treemap charts of the distribution of medical conditions in the study population. Using a standardized Medical History form during the study visit, study participants reported their medical conditions. The treemaps of these medical conditions in the pSjD group (A) and the NS-C group (B), are proportional for the number of the reported disorders. The numerical data for these treemap charts are provided in Table 1. pSjD, Sjögren's syndrome group; NS-C, non-Sjögren's syndrome control group).

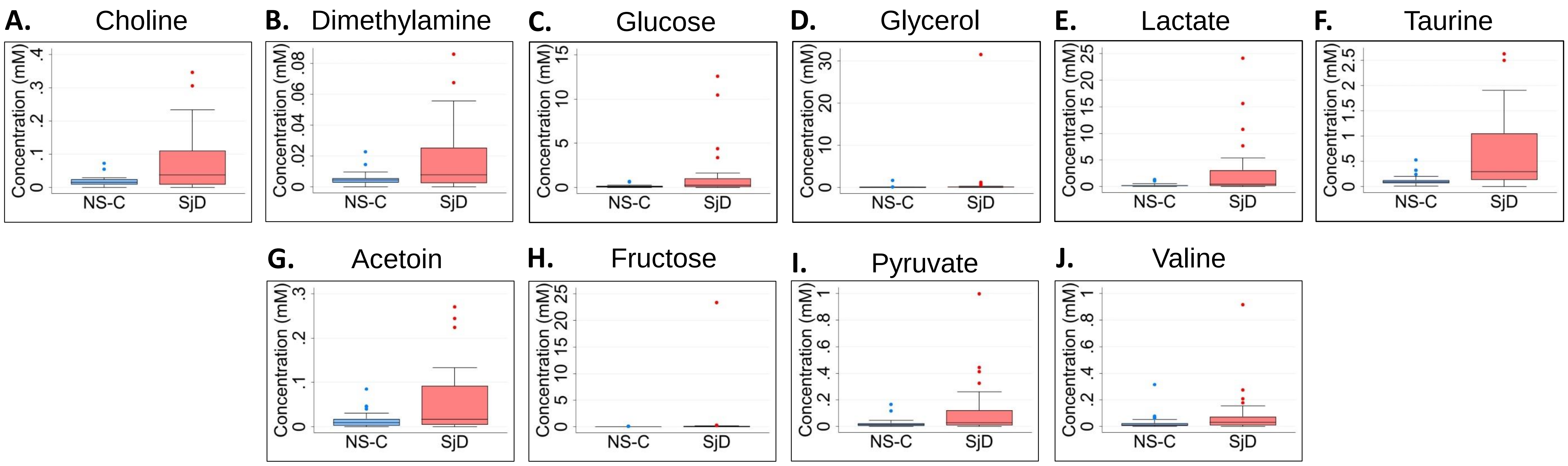

Supplementary Figure S2. Metabolite concentration comparisons (prior to p value correction) of saliva samples from pSjD and NS-C groups. When false discovery rate was not applied, the median concentration levels of choline (A), dimethylamine (B), glucose (C), glycerol (D), lactate (E), taurine (F), acetoin (G), fructose (H), pyruvate (I), and valine (J) were significantly higher in saliva samples from the pSjD group in comparison to the NS-C group. NS-C, non-Sjögren's control group (blue box plots); SjD, Sjögren's disease group (red box plots).

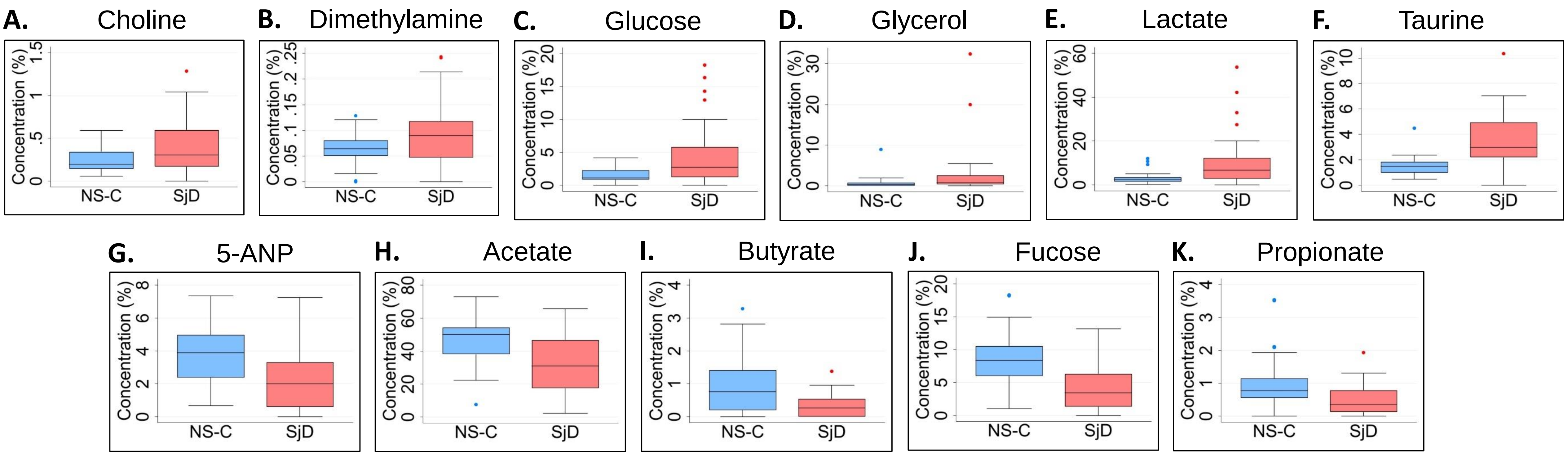

Supplementary Figure S3. Metabolite normalized data comparisons (prior to p value correction) of saliva samples from pSjD and NS-C groups. When false discovery rate was not applied, the median normalized concentration levels of choline (A), dimethylamine (B), glucose (C), glycerol (D), lactate (E), and taurine (F) were significantly higher, whereas the median concentration levels of 5-ANP (G), acetate (H), butyrate (I), fucose (J), and propionate (K) were significantly lower in saliva samples from the pSjD group in comparison to the NS-C group. NS-C, non-Sjögren's control group (blue box plots); SjD, Sjögren's disease group (red box plots); 5-ANP, 5-aminopentanoate.

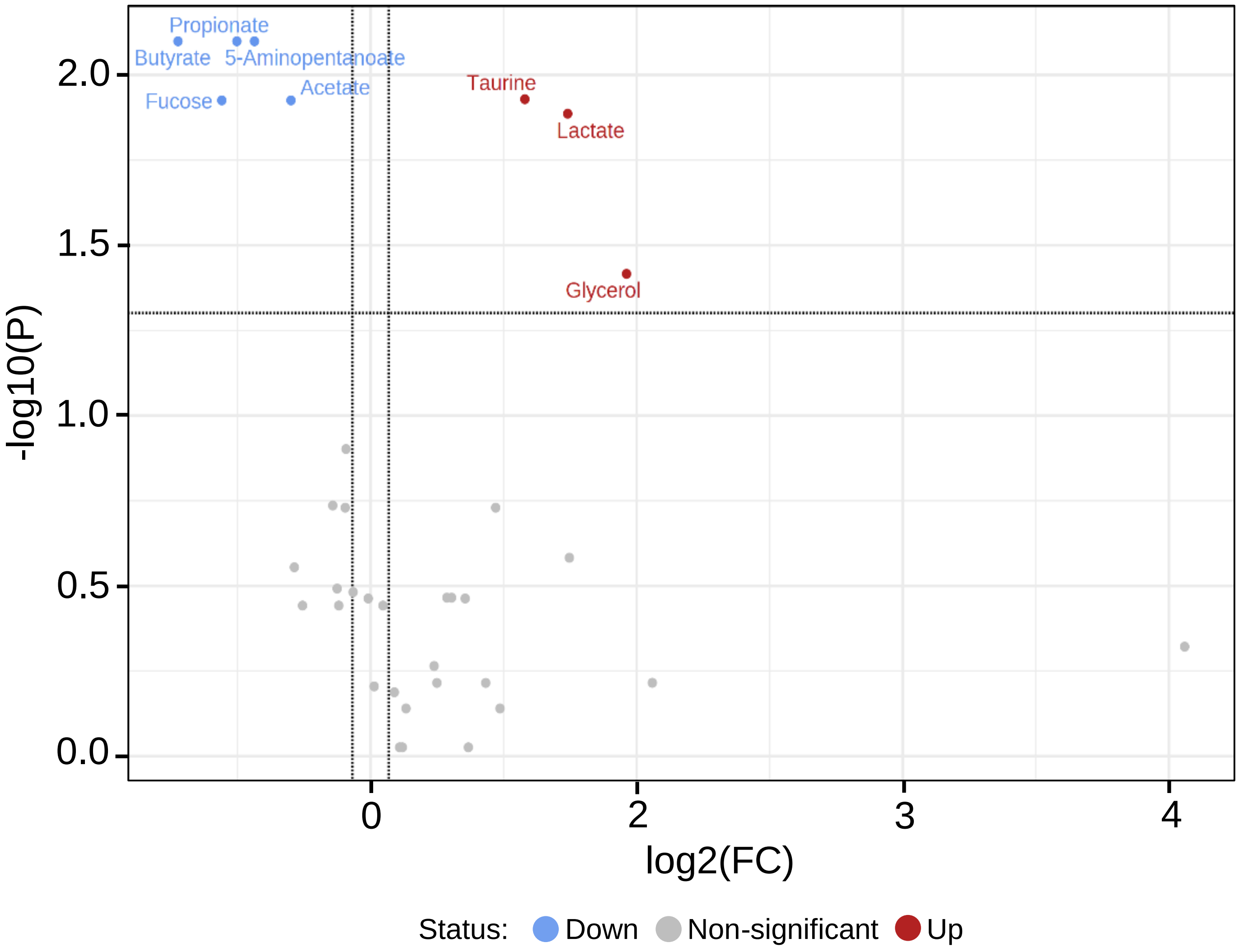

Supplementary Figure S4. Volcano plot analysis of normalized data of saliva samples from pSjD and NS-C subjects. Using Metaboanalyst Program, fold change and t-test analysis were used to compare normalized means of metabolites in pSjD versus NS-C groups. Up (red) and down (blue) refer to elevated or decreased levels of the indicated metabolites in the pSjD group, respectively. FC, fold change; pSjD, primary Sjögren's disease group; NS-C, non-Sjögren's control group.

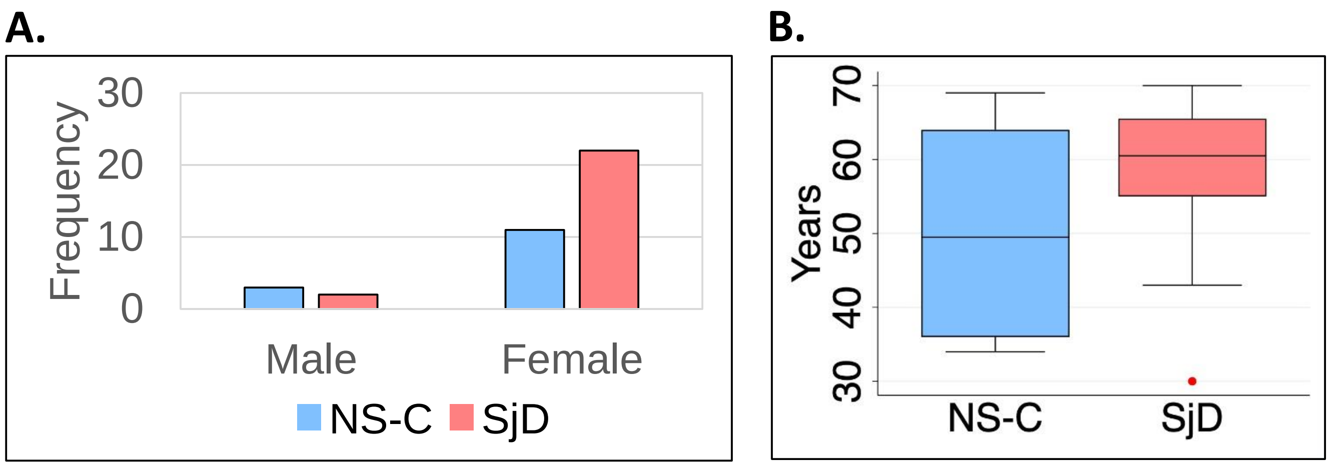

C.

Sub-group	Frequency (%)	Sex (F, M)	Age Mean (±SD)	Age P50 (iqr)	Age Min	Age Max
**NS-C**	*n*=14 (36.84%)	*n*= 11 (F) *n*= 3 (M)	50.4 (±13.7)	49.5 (28)	34	69
**pSjD**	*n*=24 (63.16%)	*n*= 22 (F) *n*= 2 (M)	59.3 (±9.1)	60.5 (10.5)	30	70
**Total**	*n*=38 (100.0%)	*n*= 33 (F) *n*= 5 (M)	56 (±11.7)	59.5 (14)	30	70

Supplementary Figure S5. Distribution of sex and age in 30-70 year old study subjects. In an age-restricted subset of subjects between 30-70 year old in this study, the pSjD sub-group (n=24) was comprised of 22 females and 2 males, while the NS-C sub-group (n=14) was comprised of 11 females and 3 males (A). The median ages of the pSjD sub-group and the NS-C sub-group were 60.5 (iqr=10.5) and 49.5 (iqr=28), respectively (B). Descriptive statistics of this subset including number, sex and age of subjects in the pSjD and NS-C groups (C). NS-C, non-Sjögren's control group; pSjD, primary Sjögren's disease group; *n,* sample size; F, female; M, male; SD, standard deviation; p50, median; iqr, interquartile range, min, minimum; max, maximum.

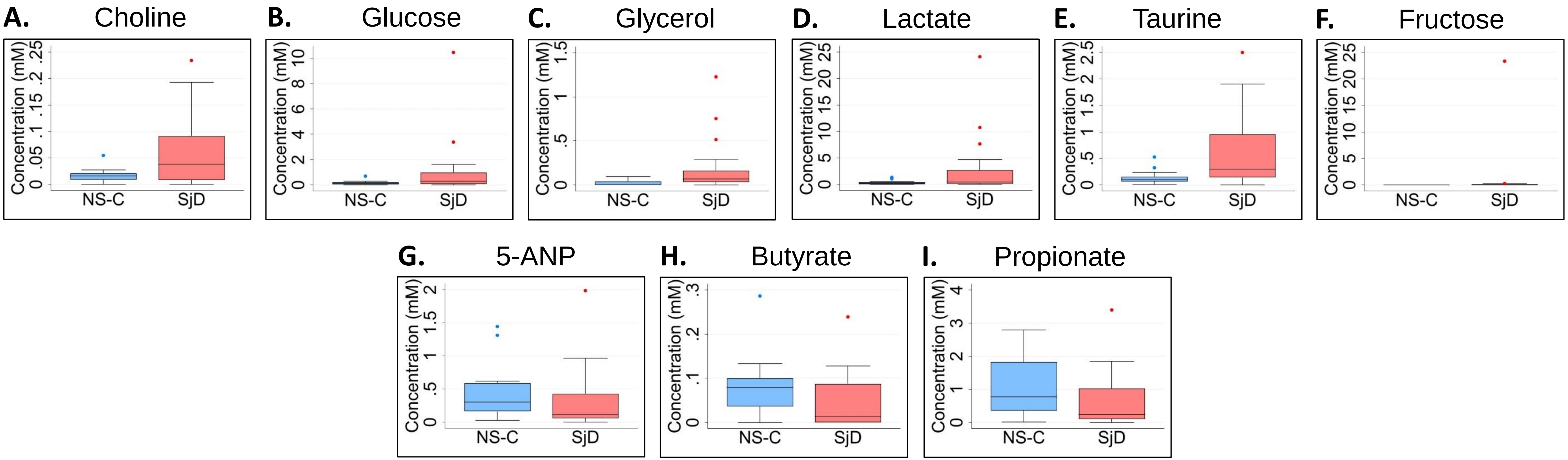

Supplementary Figure S6. Metabolite concentration comparisons (prior to p value correction) of saliva samples from a subset of age-restricted, pSjD and NS-C study participants between 30 and 70 years old. When false discovery rate was not applied, the median concentration levels of choline (A), glucose (B), glycerol (C), lactate (D), taurine (E), and fructose were significantly higher, while the median concentration levels of 5-ANP (G), butyrate (H), and propionate (I) were significantly lower in age-restricted saliva samples from the pSjD group in comparison to the NS-C group. NS-C, non-Sjögren's control group (blue box plots); SjD, Sjögren's disease group (red box plots); 5-ANP, 5-aminopentanoate.

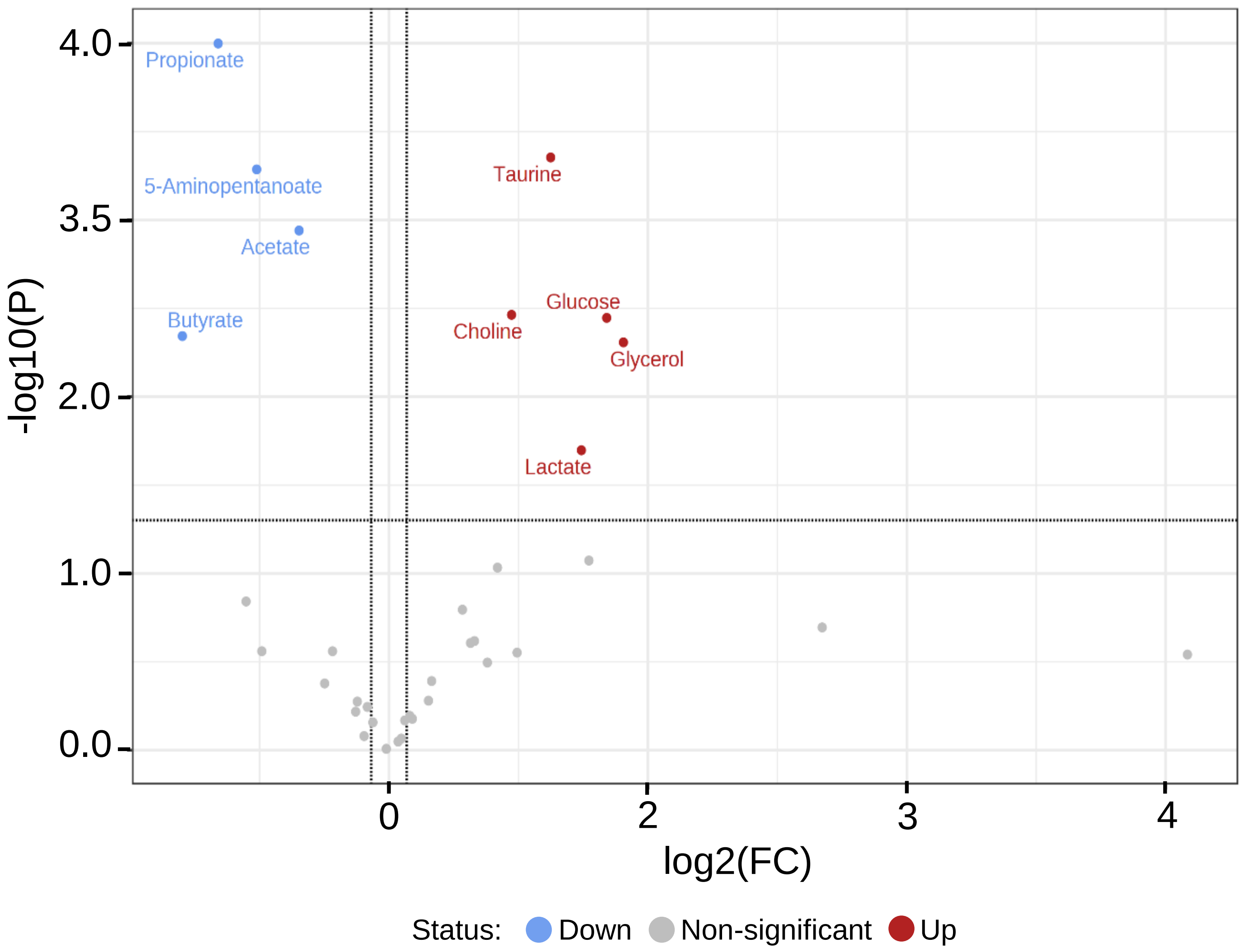

Supplementary Figure S7. Volcano plot analysis of normalized data of saliva samples from a subset of age-restricted, pSjD and NS-C study participants between 30 and 70 years old. Using Metaboanalyst Program, fold change and t-test analysis were used to compare normalized means of metabolites in NS-C versus pSjD groups. Up (red) and down (blue) refer to elevated or decreased levels in the pSjD group, respectively. FC, fold change; pSjD, primary Sjögren's disease group; NS-C, non-Sjögren's control group.

Supplementary Table S1. Demographics and clinical characteristics of the study population

Characteristic	pSjD *n*=30	NS-C *n*=30	P value
**Sex and age**			
Females, n (%)	28 (93.3%)	21 (70.0%)	0.02
Males, n (%)	2 (6.7%)	9 (30.0%)	
Females: Age (y), median (iqr)	63.7 (±8.3)	36.8 (±14.7)	< 0.001
Males: Age (y), median (iqr)	43.0 (±18.3)	38.1 (±18.4)	0.44
**Medical History reported, n (%)**			
No medical conditions	0	17 (56.7%)	< 0.001
Sjögren's	30 (100.0%)	0	< 0.001
Acid Reflux	19 (63.3%)	1 (3.3%)	< 0.001
Eye Problems	17 (56.7%)	2 (6.7%)	< 0.001
High Blood Pressure	15 (50.0%)	2 (6.7%)	< 0.001
Arthritis	15 (50.0%)	2 (6.7%)	< 0.001
High Cholesterol	9 (30%)	1 (3.3%)	0.006
Cancer	9 (30%)	0	0.002
Pain in Jaw Joints	8 (26.7%)	1 (3.3%)	0.03
Anemia	8 (26.7%)	1 (3.3%)	0.03
Immune Deficiency	8 (26.7%)	0	0.005
Thyroid Disease	8 (26.7%)	0	0.005
Chronic Headaches	7 (23.3%)	4 (13.3%)	0.32
Sinus Trouble	7 (23.3%)	0	0.01
Tonsillitis	7 (23.3%)	1 (3.3%)	0.05
Asthma	6 (20%)	1 (3.3%)	0.10
Psychological Disorders	6 (20%)	2 (6.7%)	0.25
Herpes/Cold Sores	5 (16.7%)	0	0.05
Hearing loss	5 (16.7%)	1 (3.3%)	0.20
Tumor or Growth	4 (13.3%)	0	0.11
Bronchitis	3 (10%)	0	0.24
Chest Pain	3 (10%)	0	0.24
Heart Disorder	3 (10%)	0	0.24
Neurological Disorder	3 (10%)	0	0.24
Seizures	3 (10%)	0	0.24
Head Injury	2 (6.7%)	0	0.49
Sleep Apnea	2 (6.7%)	0	0.49
Oral Lichen Planus	2 (6.7%)	0	0.49
Excessive Bleeding	2 (6.7%)	0	0.49

Liver Disease	1 (3.3%)	0	> 0.99
Radiation Therapy	1 (3.3%)	0	> 0.99
Blood Transfusion	1 (3.3%)	0	> 0.99
Diabetes	1 (3.3%)	0	> 0.99
Scarlet Fever	1 (3.3%)	1 (3.3%)	> 0.99
Joint Replacement	1 (3.3%)	0	> 0.99

pSjD, primary Sjögren's disease group; NS-C, non-Sjögren's control group.

Supplementary Table S2. Quantification of all identified metabolites in saliva samples from the study population using concentration data and normalized data

Metabolite	Metabolite Concentration (mM)		P value	Normalized Data		P value
	p50 (iqr)			p50 (iqr)		
	NS-C	pSjD		NS-C	pSjD	
5-ANP	0.218 (0.291)	0.123 (0.350)	0.060	3.893 (2.588)	2.013 (2.706)	0.0002
Acetate	3.489 (3.678)	2.620 (6.843)	0.668	50.133 (16.004)	30.961 (29.140)	0.0005
Acetoin	0.009 (0.014)	0.017 (0.088)	0.037	0.158 (0.160)	0.176 (0.238)	0.234
Alanine	0.046 (0.063)	0.095 (0.310)	0.183	0.648 (0.638)	0.647 (0.810)	0.768
Butyrate	0.061 (0.075)	0.016 (0.094)	0.068	0.766 (1.210)	0.270 (0.544)	0.0007
Choline	0.016 (0.016)	0.038 (0.102)	0.041	0.196 (0.199)	0.305 (0.426)	0.046
Citrate	0.016 (0.028)	0.033 (0.052)	0.058	0.252 (0.558)	0.398 (0.913)	0.441
Dimethyl sulfone	0.006 (0.004)	0.006 (0.009)	0.408	0.081 (0.107)	0.077 (0.093)	0.340
Dimethylamine	0.004 (0.003)	0.008 (0.023)	0.033	0.064 (0.030)	0.090 (0.071)	0.029
Ethanol	0.065 (0.054)	0.108 (0.128)	0.092	0.925 (0.706)	0.932 (1.638)	0.848
Ethanolamine	0.075 (0.083)	0.080 (0.225)	0.478	0.984 (1.144)	0.900 (0.787)	0.442
Formate	0.108 (0.215)	0.272 (0.433)	0.055	1.370 (2.411)	2.195 (4.113)	0.442
Fructose	0 (0)	0 (0.082)	0.025	0 (0)	0 (1.010)	0.059
Fucose	0.060 (0.108)	0.039 (0.103)	0.171	0.782 (0.592)	0.356 (0.657)	0.002
Glucose	0.100 (0.160)	0.277 (0.947)	0.005	1.158 (1.447)	2.760 (4.585)	0.007
Glutamine	0.040 (0.070)	0.060 (0.111)	0.495	0.531 (0.774)	0.414 (0.619)	0.178

Glycerol	0.027 (0.510)	0.071 (0.177)	0.0003	0.389 (0.803)	0.770 (2.149)	0.008
Glycine	0.107 (0.148)	0.121 (0.348)	0.574	1.975 (1.371)	1.763 (2.215)	0.768
Histidine	0.025 (0.020)	0.032 (0.070)	0.407	0.290 (0.373)	0.316 (0.440)	0.779
Lactate	0.189 (0.162)	0.432 (2.910)	0.0024	2.484 (1.984)	6.753 (9.361)	0.0013
Maltose	0.010 (0.032)	0.025 (0.148)	0.067	0.110 (0.457)	0.309 (1.430)	0.218
Methylamine	0.006 (0.007)	0.005 (0.013)	0.819	0.096 (0.094)	0.089 (0.101)	0.258
Phenylalanine	0.019 (0.015)	0.027 (0.050)	0.112	0.312 (0.273)	0.227 (0.280)	0.544
Proline	0.120 (0.164)	0.122 (0.315)	0.824	1.997 (2.190)	1.306 (1.591)	0.114
Propionate	0.535 (1.170)	0.292 (0.940)	0.183	8.368 (4.524)	3.488 (4.980)	0.0002
Pyruvate	0.013 (0.019)	0.279 (0.111)	0.025	0.198 (0.231)	0.284 (0.458)	0.151
Succinate	0.041 (0.054)	0.040 (0.220)	0.355	0.491 (0.498)	0.541 (0.551)	0.865
Sucrose	0 (0)	0 (0)	0.154	0 (0)	0 (0)	0.154
Taurine	0.100 (0.063)	0.300 (0.917)	<0.001	1.481 (0.837)	2.971 (2.751)	<0.001
Trimethylamine	0 (0.001)	0 (0.013)	0.215	0 (0.006)	0.003 (0.013)	0.338
Tyrosine	0.043 (0.040)	0.054 (0.079)	0.487	0.670 (0.455)	0.464 (0.452)	0.104
Urea	0.481 (1.040)	1.163 (1.459)	0.086	10.853 (24.440)	9.574 (30.961)	0.678
Urocanate	0 (0)	0 (0)	0.691	0 (0)	0 (0)	0.652
Valine	0.010 (0.020)	0.033 (0.065)	0.021	0.163 (0.172)	0.219 (0.304)	0.095

p50, median; iqr, interquartile range; NS-C, non-Sjögren's control group; pSjD, primary Sjögren's disease group; 5-ANP, 5-aminopentanoate.

Supplementary Table S3. P values of concentration data and normalized data of all identified metabolites in saliva samples from the study population alongside adjusted p values using Benjamini-Hochberg correction (BH) to account for false discovery rate

	**Metabolites Concentration Data (mM)**		**Metabolites Normalized Data**		**Age Restricted Metabolites Concentration Data (mM)**		**Age Restricted Metabolites Normalized Data**	
**Metabolite**	**p-value**	**Corrected p-value (BH)**	**p-value**	**Corrected p-value (BH)**	**p-value**	**Corrected p-value (BH)**	**p-value**	**Corrected p-value (BH)**
5-Aminopentanoate	0.0604	0.1580	0.0002	0.0027	0.0461	0.2411	0.0004	0.0034
Acetate	0.6681	0.7328	0.0005	0.0043	0.2732	0.4423	0.0024	0.0148
Acetoin	0.0370	0.1398	0.2338	0.3975	0.1124	0.2940	0.1432	0.3478
Alanine	0.1833	0.2968	0.7675	0.8698	0.3766	0.5820	0.5397	0.7340
Butyrate	0.0681	0.1544	0.0007	0.0048	0.0270	0.1836	0.0003	0.0041
Choline	0.0406	0.1380	0.0459	0.1419	0.0510	0.1927	0.0045	0.0191
Citrate	0.0580	0.1643	0.4413	0.6252	0.0570	0.1938	0.2105	0.4771
Dimethyl sulfone	0.4076	0.5330	0.3994	0.5904	0.1590	0.3180	0.0758	0.2148
Dimethylamine	0.0332	0.1411	0.0287	0.0976	0.1179	0.2863	0.0750	0.2318
Ethanol	0.0918	0.1836	0.8476	0.8733	0.4960	0.6746	0.7766	0.8801
Ethanolamine	0.4779	0.6018	0.4420	0.6011	0.2010	0.3797	0.0873	0.2283
Formate	0.0546	0.1688	0.4420	0.5780	0.7088	0.8310	0.9643	0.9935
Fructose	0.0254	0.1234	0.0593	0.1680	0.0160	0.1813	0.0160	0.0544
Fucose	0.1709	0.2905	0.0024	0.0117	0.7588	0.8062	0.3557	0.5497
Glucose	0.0052	0.0442	0.0068	0.0289	0.0264	0.2244	0.0040	0.0194
Glutamine	0.4955	0.5809	0.1775	0.3353	0.7239	0.8204	0.5705	0.7460
Glycerol	0.0003	0.0051	0.0080	0.0302	0.0003	0.0102	0.0024	0.0148
Glycine	0.5742	0.6508	0.7675	0.8418	0.5201	0.6801	0.6866	0.8646
Histidine	0.4071	0.5537	0.7785	0.8272	0.5547	0.6985	0.7085	0.8307
Lactate	0.0024	0.0272	0.0013	0.0074	0.0501	0.2129	0.0148	0.0559
Maltose	0.0668	0.1622	0.2180	0.3901	0.1471	0.3126	0.2550	0.4817
Methylamine	0.8186	0.8434	0.2578	0.4174	0.4055	0.5994	0.2319	0.4928
Phenylalanine	0.1119	0.2114	0.5444	0.6855	0.4497	0.6371	0.7035	0.8543
Proline	0.8244	0.8244	0.1135	0.2573	0.7085	0.8603	0.9882	0.9882
Propionate	0.1833	0.2833	0.0002	0.0027	0.0461	0.2411	0.0000	0.0003
Pyruvate	0.0253	0.1434	0.1509	0.3207	0.1064	0.3015	0.3591	0.5308
Succinate	0.3554	0.5035	0.8650	0.8650	0.9405	0.9405	0.7995	0.8495
Sucrose	0.1538	0.2752	0.1538	0.3076	0.2589	0.4633	0.2589	0.4633

Taurine	0.0002	0.0068	0.0000	0.0001	0.0057	0.0969	0.0003	0.0041
Trimethylamine	0.2147	0.3174	0.3383	0.5228	0.7349	0.8060	0.7842	0.8601
Tyrosine	0.4871	0.5915	0.1039	0.2523	0.8404	0.8659	0.2867	0.4642
Urea	0.0858	0.1823	0.6784	0.7954	0.0825	0.2550	0.3966	0.5619
Urocanate	0.6908	0.7340	0.6521	0.7918	0.2589	0.4401	0.2589	0.4401
Valine	0.0206	0.1401	0.0947	0.2477	0.1415	0.3207	0.2321	0.4642
